# RAFT Dispersion Polymerization of 2-Hydroxyethyl Methacrylate in Non-polar Media

**DOI:** 10.1021/acs.macromol.4c02016

**Published:** 2024-12-04

**Authors:** Priyanka Chohan, Csilla György, Oleksandr O. Mykhaylyk, Giles M. Prentice, Sorin V. Filip, Marc J. Payne, Gouranga Manna, Steven P. Armes

**Affiliations:** † Dainton Building, Department of Chemistry, University of Sheffield, Brook Hill, Sheffield, South Yorkshire S3 7HF, U.K.; ‡ Applied Sciences, BP Technology Centre, Whitchurch Hill, Reading RG8 7QR, U.K.; § European Synchrotron Radiation Facility, 6 rue Jules Horowitz, Grenoble 38000, France

## Abstract

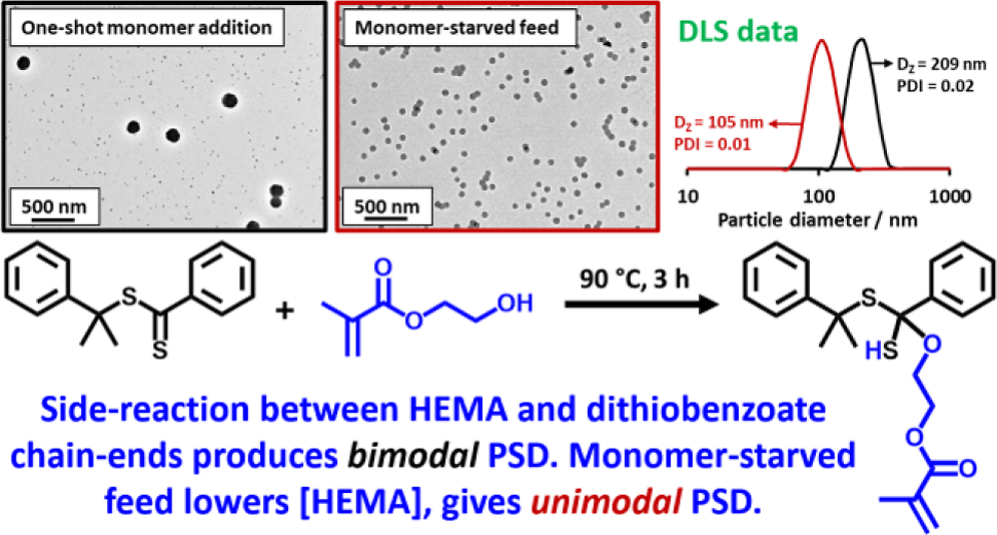

We report the reversible addition–fragmentation chain transfer (RAFT) dispersion polymerization of 2-hydroxyethyl methacrylate (HEMA) in *n*-dodecane using a poly(lauryl methacrylate) (PLMA) precursor at 90 °C. This formulation is an example of polymerization-induced self-assembly (PISA), which leads to the formation of a colloidal dispersion of spherical PLMA–PHEMA nanoparticles at 10–20% w/w solids. PISA syntheses involving polar monomers in non-polar media have been previously reported but this particular system offers some unexpected and interesting challenges in terms of both synthesis and characterization. First, GPC analysis requires chemical derivatization of the pendent hydroxyl groups in the PHEMA block using excess acetyl chloride to ensure that both blocks are fully soluble in chloroform. Second, DLS, TEM and ^1^H NMR spectroscopy studies of the periodically sampled polymerizing mixture indicate the transient formation of anomalously large, colloidally unstable aggregates at around 50% conversion, which approximately corresponds to the maximum rate of polymerization. Remarkably, such aggregates immediately break up to form well-defined nanoparticles, which remain colloidally stable at the end of the HEMA polymerization. Moreover, depending on the target degree of polymerization (DP) for the PHEMA block, TEM studies typically indicate bimodal particle size distributions for PLMA–PHEMA nanoparticles prepared using a one-shot batch protocol. This is attributed to a side-reaction between HEMA monomer and the dithiobenzoate-based RAFT agent. Fortunately, this problem can be prevented by conducting such PISA syntheses under monomer-starved conditions by continuous addition of the HEMA monomer using a syringe pump. Alternatively, unimodal spheres can also be produced via adding HEMA in multiple batches. This PISA formulation has been optimized to produce monomodal particle size distributions while targeting a PHEMA DP of up to 1000 at the maximum possible copolymer concentration. Finally, time-resolved small-angle X-ray scattering (SAXS) studies indicate the rapid formation of well-defined near-monodisperse spheres when targeting PLMA_14_–PHEMA_50_ nanoparticles.

## Introduction

Polymerization-induced self-assembly (PISA) has become well-established as a powerful and versatile technique for the efficient synthesis of a wide range of block copolymer nanoparticles.^1−7^ PISA involves the chain extension of a soluble polymer precursor using a vinyl monomer in a suitable solvent that is chosen to be a non-solvent for the growing second block. As this insoluble block grows, micellar nucleation occurs via self-assembly at some critical mean degree of polymerization (DP). The nascent nuclei quickly become monomer-swollen, which typically leads to a significant rate acceleration.^8,9^ The remaining monomer is almost fully consumed within a few hours to produce a colloidal dispersion of sterically stabilized nanoparticles, with the initial soluble polymer acting as the steric stabilizer. PISA can be conducted using many different types of either vinyl or cyclic monomers in a wide range of either polar or non-polar solvents.^7,10−27^ The final copolymer morphology is often spheres, but targeting a higher volume fraction of the insoluble block can often provide convenient access to highly anisotropic worms or polydisperse vesicles.^8,28−36^

The first examples of PISA in non-polar media were reported by Charleux and co-workers,^37−39^ who also filed a cosmetics application with L’Oreal based on such formulations.^40^ Subsequently, Fielding et al. reported the first pseudophase diagram for non-polar PISA syntheses,^15,29,41^ which aids the reproducible targeting of pure copolymer morphologies such as worms^42^ or vesicles.^43^ In the case of spheres, time-resolved small-angle X-ray scattering (SAXS) has been used to study the evolution of particle size over time.^44^ In a recent follow-up study, direct evidence was obtained for monomer-swollen nascent nanoparticles to support the so-called “nanoreactor” concept that is often invoked for PISA syntheses.^9^ Spherical block copolymer nanoparticles have also been examined as nanoparticle lubricants for automotive engine oils^45^ and the introduction of epoxy functional groups has been demonstrated to enhance their chemical adsorption onto stainless steel.^46^ In addition, diblock copolymer worms prepared directly in silicone oil can serve as a viscosity modifier.^47^ Perrier and co-workers reported the synthesis of graft copolymer nanoparticles in non-polar media.^48^ All of the above syntheses involve reversible addition–fragmentation chain transfer (RAFT) polymerization^49,50^ but anionic polymerization,^51−55^ group transfer polymerization (GTP)^56,57^ or ring-opening polymerization can also be utilized.^58−61^

**Scheme 1:**
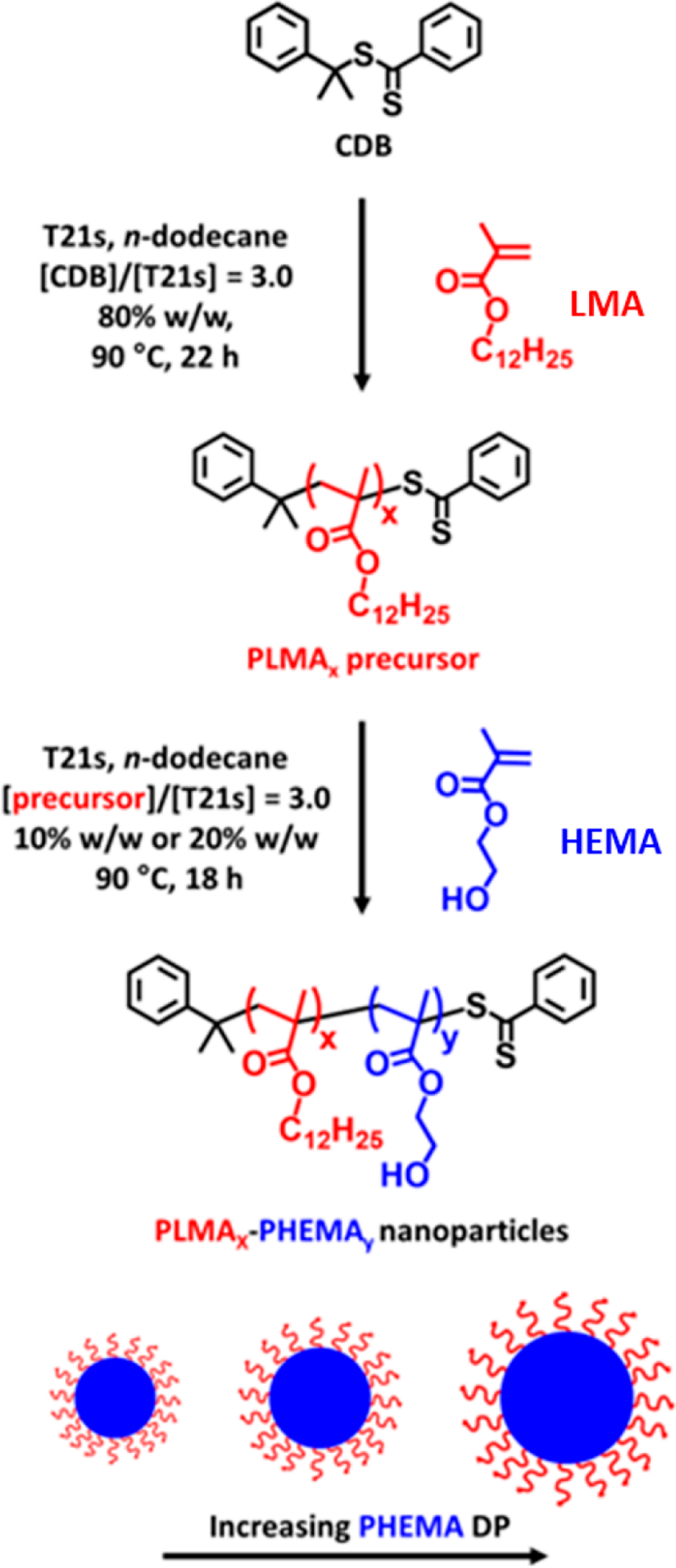
One-Pot Synthesis of a Poly(lauryl methacrylate) (PLMA) Precursor via RAFT Solution Polymerization in *n*-Dodecane Using Cumyl Dithiobenzoate (CDB) at 90 °C, Immediately Followed by the RAFT Dispersion Polymerization of 2-Hydroxyethyl Methacrylate (HEMA) in *n*-Dodecane at 90 °C

For PISA syntheses conducted in non-polar media, the insoluble structure-directing block is usually based on a non-polar monomer such as methyl methacrylate (MMA) or benzyl methacrylate (BzMA).^30,33,62^ However, there are also a few literature examples of the use of polar vinyl monomers such as *N*-2-(methacryloyloxy)ethyl pyrrolidone (NMEP),^63^*N*-2-(acryloyloxy)ethyl pyrrolidone (NAEP),^64^ 2-hydroxypropyl methacrylate (HPMA)^32^ or glycidyl methacrylate (GlyMA).^65^ The rate of polymerization tends to be much faster for such formulations, which is most likely the result of stronger monomer partitioning within the nascent growing nanoparticles.^9^ Herein we report the RAFT dispersion polymerization of a relatively polar monomer, 2-hydroxyethyl methacrylate (HEMA), in *n*-dodecane using a poly(lauryl methacrylate) (PLMA) precursor (see Scheme 1). This particular PISA formulation presents some unusual and unexpected challenges in terms of both synthesis and characterization. We identify appropriate solutions to these technical problems while optimizing such syntheses to obtain well-defined spherical nanoparticles of tunable diameter at the highest possible copolymer concentration. Time-resolved small-angle X-ray scattering (SAXS) is also used to monitor the evolution in copolymer morphology for one of these PISA formulations.

## Experimental Section

### Materials

Lauryl methacrylate (LMA; 96%) was purchased from Sigma-Aldrich (UK) and filtered through basic alumina to remove monomethyl ether hydroquinone (MEHQ) inhibitor prior to use. 2-Hydroxyethyl methacrylate (HEMA, triply distilled ULTRA grade; 0.1 mol % dimethacrylate impurity) was kindly provided by GEO Specialty Chemicals (Hythe, UK) and was used as received. Cumyl dithiobenzoate (CDB; 99%) and acetyl chloride (98%) were purchased from Sigma-Aldrich (UK) and were used as received. Chloroform, *n*-dodecane and *d*_5_-pyridine were purchased from Thermo Fisher Scientific (UK). *d*_2_-Dichloromethane (CD_2_Cl_2_) was purchased from Cambridge Isotope Laboratory (USA) and *tert*-butyl peroxy-2-ethylhexanoate (T21s) initiator was supplied by AkzoNobel (The Netherlands).

### Synthesis of PLMA_*x*_ Precursor via RAFT Solution Polymerization of LMA in *n*-Dodecane

PLMA_14_ and PLMA_196_ precursors were prepared at 80% w/w solids by adapting a previously reported protocol.^30^ The synthesis of the PLMA_14_ precursor was conducted as follows. LMA (1.40 g; 5.507 mmol), CDB (0.10 g; 0.367 mmol; target DP = 15), T21s initiator (26.5 mg; 0.122 mmol; CDB/T21s molar ratio = 3.0; dissolved at 10% v/v in *n*-dodecane) and *n*-dodecane (0.382 g) were weighed into a 14 mL glass vial, which was sealed using a rubber septum. The resulting solution was purged with nitrogen gas for 30 min. Then the sealed vial was immersed in a preheated oil bath at 90 °C with continuous stirring for 22 h. A final LMA conversion of 95% was determined by ^1^H NMR spectroscopy studies in CD_2_Cl_2_ by comparing the integrated vinyl monomer signals at 5.6 and 6.1 ppm to the two oxymethylene protons assigned to PLMA at 3.8–4.1 ppm. Hence the mean DP of this crude PLMA precursor was calculated to be approximately 14. Chloroform GPC analysis using a refractive index detector and a series of near-monodisperse poly(methyl methacrylate) calibration standards indicated an *M*_n_ of 5500 g mol^–1^ and an *M*_w_/*M*_n_ of 1.19. Similarly, a PLMA_196_ precursor was also prepared (target DP = 200). A final LMA conversion of 98% was determined by ^1^H NMR spectroscopy. In this case, chloroform GPC analysis (refractive index detector) indicated an *M*_n_ of 35,300 g mol^–1^ and an *M*_w_/*M*_n_ of 1.21.

### Synthesis of PLMA_*x*_–PHEMA_*y*_ Nanoparticles via RAFT Dispersion Polymerization of HEMA Using a Single Batch One-Shot Protocol

A series of PLMA_14_–PHEMA_*y*_ (*y* = 25–149) nanoparticles at 20% w/w solids and a series of PLMA_196_–PHEMA_*y*_ (*y* = 95–990) nanoparticles at 10% w/w solids were prepared via a one-pot protocol via the one-shot addition of HEMA monomer. A typical synthesis of PLMA_14_–PHEMA_25_ spherical nanoparticles prepared at 20% w/w solids was conducted as follows. Unpurified PLMA_14_ precursor (0.213 g, 80% w/w solids; 43.8 μmol), HEMA monomer (0.142 g; 1.09 mmol; target DP = 25), T21s initiator (3.16 mg; 14.6 μmol; dissolved at 10% v/v in *n*-dodecane) and *n*-dodecane (1.22 g) were weighed into a 14 mL glass vial and purged with nitrogen gas for 30 min. The vial was then immersed in a preheated oil bath at 90 °C and the reaction mixture was magnetically stirred for 18 h. ^1^H NMR analysis in *d*_5_-pyridine indicated that full HEMA monomer conversion was achieved (the integrated monomer vinyl signal at 5.45 ppm was compared to the hydroxyl signal assigned to PHEMA at 6.3–6.7 ppm). After derivatization of this PLMA_14_–PHEMA_25_ diblock copolymer using excess acetyl chloride, chloroform GPC analysis (refractive index detector) indicated an *M*_n_ of 10,200 g mol^–1^ and an *M*_w_/*M*_n_ of 1.31. Relatively high HEMA conversions (≥98%) were achieved for all diblock copolymer syntheses conducted at 20% w/w solids using the PLMA_14_ precursor (see Table S1). Such copolymers were subjected to chloroform GPC analysis after derivatization. Similarly, relatively high HEMA (≥95%) conversions could be achieved for all diblock copolymer syntheses conducted at 10% w/w solids using the PLMA_196_ precursor (see Table S2). However, the latter copolymers could not be analyzed via chloroform GPC owing to their relatively high degree of core cross-linking.

### Derivatization of PLMA_14_–PHEMA_*y*_ Nanoparticles with Acetyl Chloride

A series of PLMA_14_–PHEMA_*y*_ nanoparticles (*y* = 25–149) were derivatized to enable chloroform GPC analysis. A typical protocol for the functionalization of PLMA_14_–PHEMA_25_ spheres with acetyl chloride was conducted as follows. In a 7 mL glass vial, acetyl chloride (14.8 μL, 0.208 mmol; [CH_3_COCl]/[HEMA] molar ratio = 1.5) was added to 0.20 g of a 20% w/w dispersion of PLMA_14_–PHEMA_25_ spheres in *n*-dodecane via micropipette and the reaction mixture was magnetically stirred at 25 °C for 19 h. The degree of esterification was determined by monitoring the appearance of the acetylated HEMA signal at 2.1 ppm using ^1^H NMR spectroscopy. Such derivatization led to molecular dissolution of the diblock copolymer chains in common organic solvents such as chloroform.

### Reaction of CDB RAFT Agent with HEMA Monomer in the Absence of Any Initiator

CDB RAFT agent (0.080 g, 0.294 mmol) was mixed with HEMA monomer (0.382 g, 2.94 mmol; [HEMA]/[CDB] molar ratio = 10) in a 7 mL glass vial before being transferred to an NMR tube. *d*_2_-Dichloromethane was then placed in an inner NMR tube within the mixture to be used as a solvent lock and an initial ^1^H NMR spectrum was recorded. The inner NMR tube was then removed and the 10:1 HEMA/CDB mixture was heated for 3 h at 90 °C in a preheated oil bath with continuous stirring. Then the reaction mixture was cooled to 25 °C and a second ^1^H NMR spectrum was recorded using the same solvent lock. Liquid chromatography–mass spectrometry (LC–MS) analysis was also conducted on the same 10:1 HEMA/CDB mixture.

### Centrifugal Separation of Small and Large PLMA_196_–PHEMA_990_ Nanoparticles

A 9.9% w/w dispersion of PLMA_196_–PHEMA_990_ nanoparticles prepared by the one-shot method in *n*-dodecane was weighed into a preweighed 1.5 mL microfuge tube (0.774 g). This tube was placed into a balanced microcentrifuge (Heraeus Biofuge Pico D-37520) and centrifugation was conducted for 20 min at 13,000 rpm, after which the supernatant (containing the relatively small nanoparticles, *D*_*z*_ = 64 nm) was removed. The sediment (consisting of the relatively large nanoparticles, *D*_*z*_ = 211 nm) was placed in a vacuum oven set at 50 °C and dried to constant mass. A solids content of 8.5% w/w was determined for the relatively large nanoparticles. By difference, a solids content of 1.4% w/w was estimated for the relatively small nanoparticles.

### Synthesis of PLMA_196_–PHEMA_1000_ Nanoparticles via RAFT Dispersion Polymerization of HEMA by Addition of Sequential Multiple Monomer Batches

PLMA_196_–PHEMA_1000_ nanoparticles were targeted at 10% w/w solids by adding HEMA monomer in two, four or eight equal batches. A typical example of HEMA addition in two equal batches was conducted as follows. The unpurified PLMA_196_ precursor (0.250 g, 80% w/w solids; 3.97 μmol), T21s initiator (0.287 mg; 1.32 μmol; dissolved at 10% v/v in *n*-dodecane), *n*-dodecane (6.41 g) and the first batch of HEMA monomer (0.259 g; 1.99 mmol; target DP = 500) were added to a 14 mL glass vial, which was sealed and then purged using nitrogen gas for 30 min. The sealed vial was then placed in a preheated oil bath set at 90 °C with continuous magnetic stirring. After 30 min, HEMA (∼0.40 g) was placed in a separate 14 mL glass vial and purged using nitrogen gas for 30 min. The second batch of degassed HEMA (0.259 g, 1.99 mmol; target DP = 500) was then added to the reaction mixture via syringe and the second-stage polymerization was allowed to proceed for 20 h at 90 °C. A final HEMA conversion of 99% was determined by ^1^H NMR analysis in *d*_5_-pyridine, indicating a mean PHEMA DP of 990. The four-batch and eight-batch HEMA addition syntheses were conducted in a similar manner with each HEMA batch added at 1 h intervals, resulting in final HEMA conversions of 98% (with a mean PHEMA DP of 980) for the four-batch synthesis and 93% (with a mean PHEMA DP of 930) for the eight-batch synthesis (see Table S3).

### Synthesis of PLMA_196_–PHEMA_1000_ Nanoparticles via RAFT Dispersion Polymerization of HEMA under Monomer-Starved Conditions

PLMA_196_–PHEMA_1000_ nanoparticles were targeted at 10% w/w solids via monomer-starved addition of HEMA. Unpurified PLMA_196_ precursor (0.500 g, 80% w/w solids; 7.95 μmol), T21s initiator (0.573 mg; 2.65 μmol; dissolved at 10% v/v in *n*-dodecane) and *n*-dodecane (12.81 g) were weighed into a 28 mL glass vial. HEMA monomer (1.5 g) was placed in a separate 14 mL glass vial. Both vials were then sealed with rubber septa and purged with nitrogen gas for 30 min. Degassed HEMA monomer (1.03 g; 7.95 mmol; target DP = 1000) was drawn into a 2.0 mL plastic syringe, which was then placed in a motorized Aladdin AL-1000 syringe pump. The glass vial containing the PLMA_196_ precursor was immersed in a preheated oil bath set at 90 °C, and HEMA monomer was added dropwise over 1 h at a rate of 0.96 mL h^–1^ with continuous stirring. After complete addition of the monomer, the polymerization was allowed to proceed for a further 3 h at 90 °C. A final HEMA conversion of 98% was determined via ^1^H NMR analysis in *d*_5_-pyridine, indicating a mean PHEMA DP of 980. Similarly, PLMA_196_–PHEMA_400_ nanoparticles were targeted at 10% w/w solids using the same rate of monomer addition (0.96 mL h^–1^). A final HEMA conversion of 99% was achieved, indicating a mean PHEMA DP of 396 (see Table S4).

### ^1^H NMR Spectroscopy

^1^H NMR spectra were recorded in either CD_2_Cl_2_ or *d*_5_-pyridine using a 400 MHz Bruker Avance spectrometer. Typically, 64 scans were averaged per spectrum.

### Gel Permeation Chromatography (GPC)

Chloroform GPC was used to assess the molecular weight distributions (MWDs) of the PLMA_14_ and PLMA_196_ precursors, plus a series of PLMA_14_–PHEMA_*y*_ diblock copolymers. The instrumental setup comprised an Agilent 1260 GPC system, two Agilent PL gel 5 μm Mixed C columns which were connected in series with a guard column, 1260 Infinity II refractive index detector and 1260 Infinity II variable wavelength detector. The eluent contained 0.25% TEA by volume, the operating temperature was 40 °C and the flow rate was 1.0 mL min^–1^. A series of eight near-monodisperse poly(methyl methacrylate) standards (*M*_p_ values ranging from 800 to 988,000 g mol^–1^) was used for calibration.

### Dynamic Light Scattering (DLS)

DLS studies were performed using a Zetasizer Nano ZS instrument (Malvern Instruments, UK) at a fixed scattering angle of 173°. Copolymer dispersions were diluted to 0.10% w/w using *n*-dodecane prior to light scattering studies at 25 °C. The *z*-average diameter and DLS polydispersity were calculated by cumulant analysis of the experimental correlation function using Dispersion Technology Software version 6.20. Data were averaged over three consecutive runs consisting of ten measurements per run.

### Transmission Electron Microscopy (TEM)

TEM studies were conducted using a FEI Tecnai G2 Spirit instrument operating at 80 kV and equipped with a Gatan 1k CCD camera. A single droplet of a 0.10% w/w copolymer dispersion was placed onto a carbon-coated copper grid and allowed to dry, prior to exposure to ruthenium(VIII) oxide vapor for 7 min at 20 °C.^66^ This heavy metal compound acted as a positive stain for the core-forming PHEMA block to improve electron contrast. The ruthenium(VIII) oxide was prepared as follows: ruthenium(IV) oxide (0.30 g) was added to water (50 g) to form a black slurry; subsequent addition of sodium periodate (2.0 g) with continuous stirring produced a yellow solution of ruthenium(VIII) oxide within 1 min at 20 °C.

### LC–MS Analysis

This measurement was conducted using an Agilent 1260 Infinity LC instrument and Agilent 6530 Q-ToF MS instrument equipped with a 1.8 μm Agilent Zorbax Extend-C18 2.1 mm × 50 mm column and a mobile phase comprising two solvents (A = water + 0.1% formic acid and B = acetonitrile + 0.1% formic acid). The solvent gradient was switched from 5% B to 95% B over 15 min at a flow rate of 0.40 mL min^–1^ using an injection volume of 1.0 μL. The mass spectrometer was configured in its ESI positive ion mode over an *m*/*z* range of 100–2400 using a drying gas temperature of 350 °C, a capillary voltage of 4000 V, and a drying gas flow rate of 11 L min^–1^.

### Small-Angle X-ray Scattering (SAXS)

SAXS experiments were conducted on four 1.0% w/w dispersions of PLMA_14_–PHEMA_25–119_ nanoparticles in *n*-dodecane at the ESRF (station ID02, Grenoble, France) using monochromatic X-ray radiation (λ = 1.03 A; *q* range = 0.04 to 3.9 nm^–1^, where *q* is the length of the scattering vector and θ is one-half of the scattering angle, such that *q* = 4π sin θ/λ) and a Eiger2 4M two-dimensional detector (Dectris, Switzerland). A flow-through glass capillary of 1.47 mm diameter was used as a sample holder. Scattering data (including a correction for secondary scattering contributions from the beamline windows^67^) were reduced using standard routines provided by the beamline, and were further analyzed using Irena SAS macro for Igor Pro.^68^

A time-resolved SAXS study was also performed at station ID02. In this case, a 20% w/w dispersion of PLMA_14_–PHEMA_50_ nanoparticles was targeted in *n*-dodecane. The experimental setup was as follows. Unpurified PLMA_14_ precursor (1.375 g, 80% w/w solids; 0.277 mmol), HEMA monomer (1.805 g; 13.9 mmol; target DP = 50), T21s initiator (20.0 mg; 92.5 μmol; dissolved at 10 v/v in *n*-dodecane) and *n*-dodecane (11.4 g) were charged into a 50 mL round-bottomed flask, sealed and purged with nitrogen gas for 30 min. With the outlet needle and nitrogen gas inlet needle still inserted, a third needle connected to Teflon tubing (16.5 cm length, 0.50 mm diameter) was inserted into the reaction flask. The other end of the tubing was connected to the same 1.47 mm diameter flow-through cell set at 80 °C, which is the upper limit temperature for this setup. This cell was connected to a motorized Aladdin AL-1000 syringe pump by Teflon tubing. The reaction flask was then immersed in a preheated oil bath set at 90 °C and the reaction mixture was magnetically stirred. The syringe pump was used to withdraw the reaction mixture at a rate of 0.50 mL min^–1^ for 30 min. This setup had a dead time of 2.37 min, which corresponds to the time taken for the reaction mixture to first reach the flow-through cell (see Figure S1). In principle, the intense synchrotron X-ray beam could enhance the rate of polymerization by generating an additional radical flux.^44,69^ However, this potential problem—plus the possibility of in situ beam damage—was avoided by continuously flowing the reaction mixture through the capillary cell during the TR-SAXS experiment.

## Results and Discussion

### Synthesis of PLMA_14_–PHEMA_*y*_ Nanoparticles at 20% w/w Solids Using a One-Shot One-Pot Protocol

**Scheme 2:**
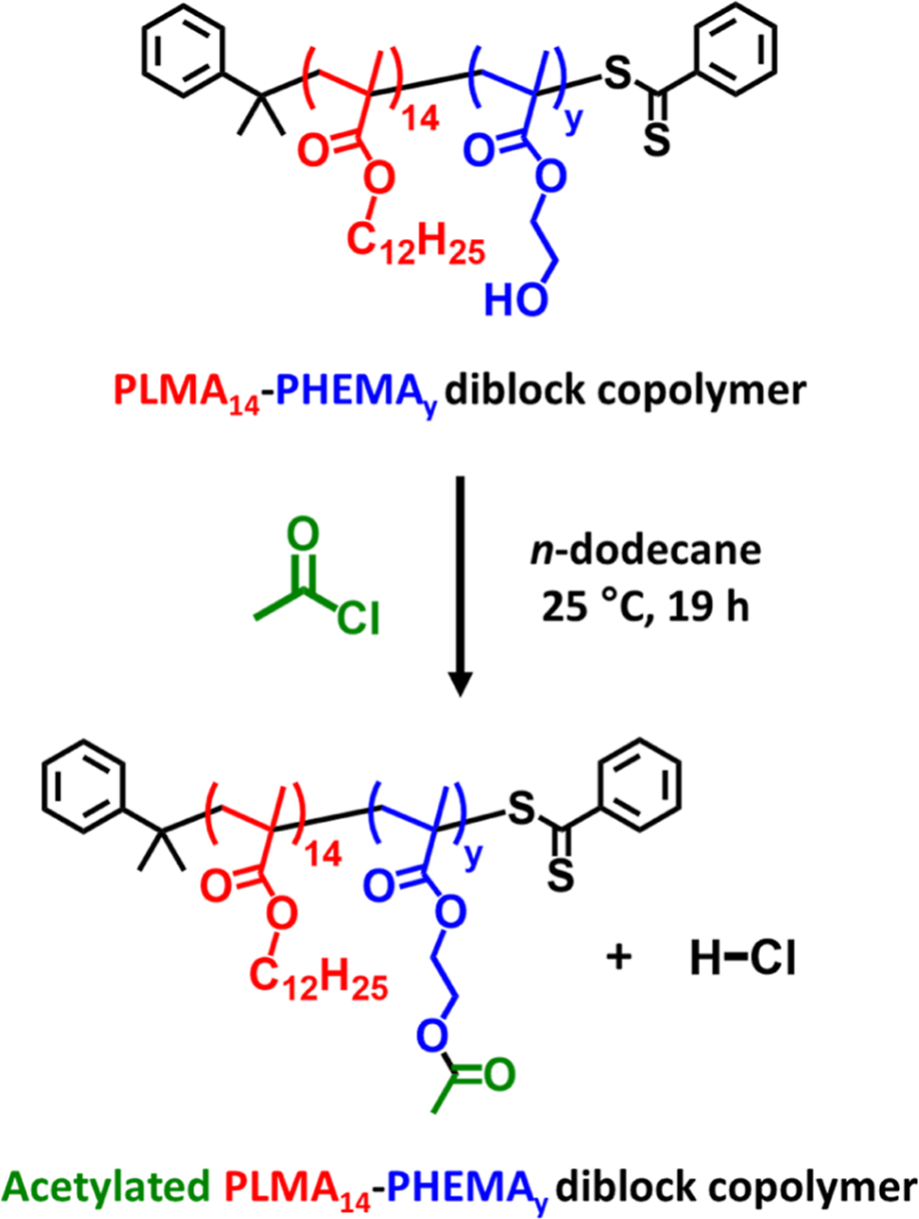
Esterification of PLMA_14_–PHEMA_*y*_ Diblock Copolymer in *n*-Dodecane at 25 °C Using Excess Acetyl Chloride ([CH_3_COCl]/[HEMA] Molar Ratio = 1.5) to Enable Chloroform GPC Analysis

A PLMA_14_ precursor was synthesized via RAFT solution polymerization of LMA at 80% w/w solids in *n*-dodecane at 90 °C using CDB as a RAFT agent (see Scheme 1). Without any purification, this PLMA_14_ precursor was then chain-extended with HEMA to target PLMA_14_–PHEMA_*y*_ nanoparticles (where *y* = 25–149) via RAFT dispersion polymerization of HEMA at 20% w/w solids. Unfortunately, the resulting copolymer chains were not soluble in any common GPC solvent so chemical derivatization was required to assess the chain extension efficiency and the molecular weight distribution. This involved esterification of the hydroxylated polymer by addition of excess acetyl chloride ([CH_3_COCl]/[HEMA] = 1.5) to a 20% w/w dispersion of PLMA_14_–PHEMA_*y*_ nanoparticles at 25 °C. After reaction for 19 h, the resulting acetylated diblock copolymer chains were fully soluble in chloroform (see Scheme 2). The degree of esterification was determined to be approximately 100% as judged by ^1^H NMR analysis, see Figure S2.

**Figure 1:**
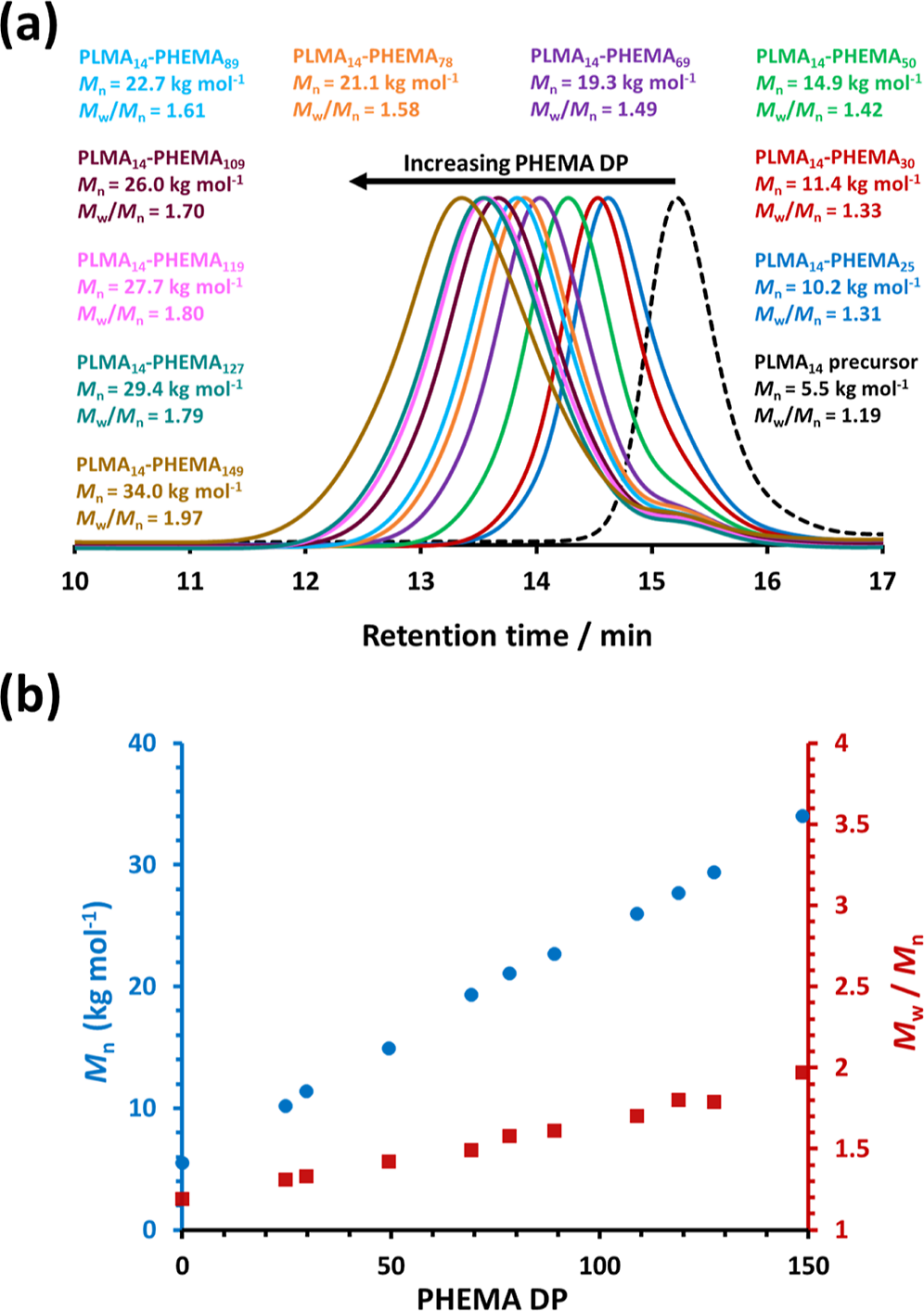
(a) Chloroform GPC curves [vs a series of near-monodisperse poly(methyl methacrylate) calibration standards, which incur a systematic error in the *M*_n_ data] recorded using a refractive index detector for a PLMA_14_ precursor (black dashed curve) prepared by RAFT solution polymerization of LMA in *n*-dodecane at 80% w/w solids, and ten corresponding PLMA_14_–PHEMA_*y*_ diblock copolymers prepared by RAFT dispersion polymerization of HEMA in *n*-dodecane at 90 °C when targeting 20% w/w solids and y = 25 – 150. (b) Linear relationship between GPC *M*_n_ (blue circles) and actual PHEMA DP (as determined by ^1^H NMR studies) observed for the same series of PLMA_14_–PHEMA_*y*_ diblock copolymers. The corresponding *M*_w_/*M*_n_ (red squares) data are also shown.

Chromatograms recorded for ten PLMA_14_–PHEMA_25–149_ diblock copolymers using a refractive index detector are shown in Figure 1a, along with the corresponding chromatogram recorded for the PLMA_14_ precursor. The latter had a relatively narrow MWD (*M*_w_/*M*_n_ = 1.19) compared to the diblock copolymer MWDs (1.31 ≤ *M*_w_/*M*_n_ ≤ 1.97). Increasing the target PHEMA DP from 25 to 149 led to the appearance of a low molecular weight shoulder, which is assigned to the PLMA_14_ precursor. Despite this feature, the chain extension efficiency appears to be reasonably high.

Moreover, there is a linear correlation between the GPC *M*_n_ data and the actual PHEMA DP (after correcting for each HEMA conversion) when the latter is systematically increased from 25 to 149 (see Figure 1b).^29,32,70^ As expected, increasing the PHEMA DP led to a gradual increase in the breadth of the MWD. For example, an *M*_w_/*M*_n_ of 1.97 was observed when targeting a PHEMA DP of 150.

**Figure 2:**
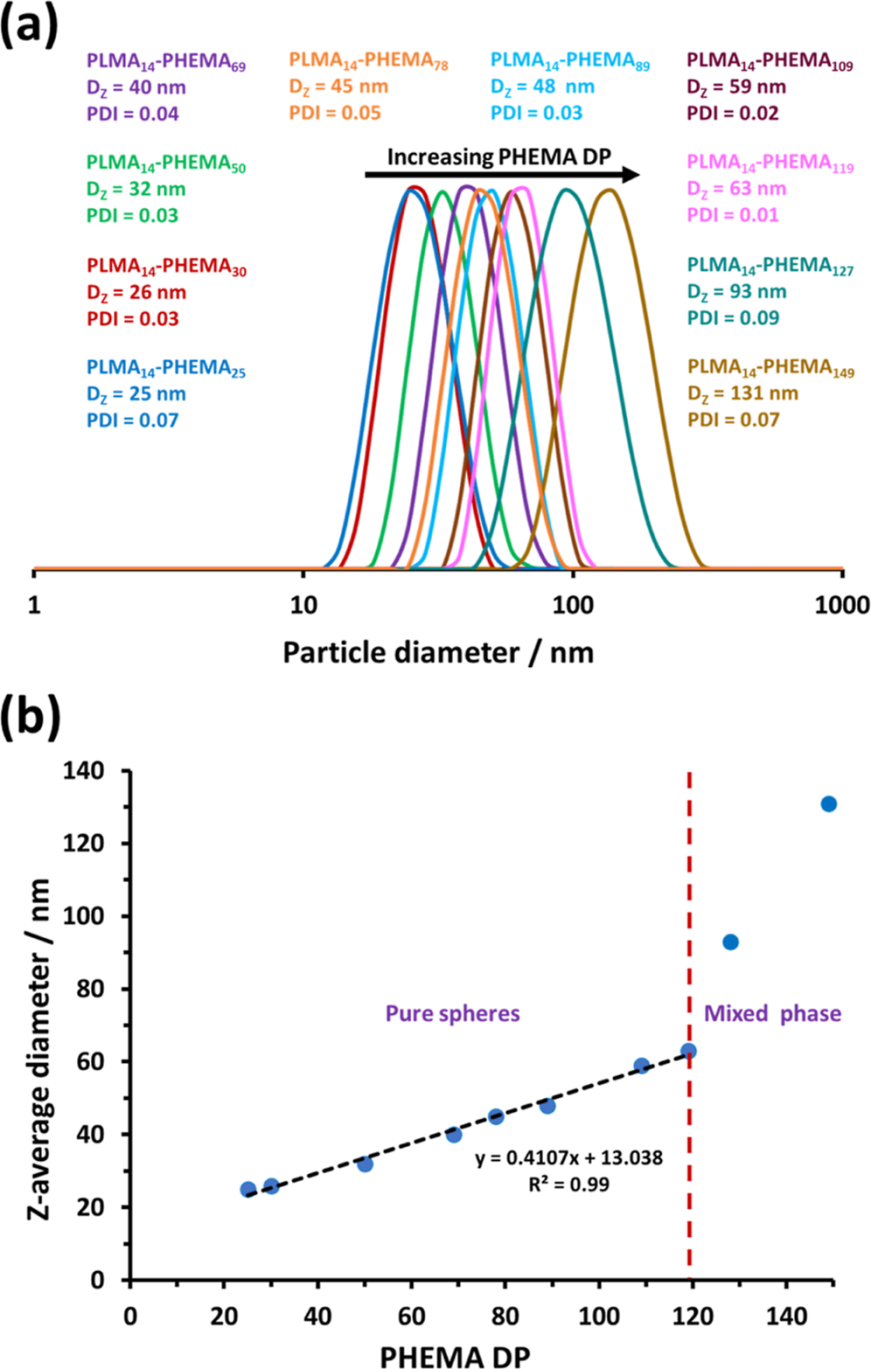
(a) DLS particle size distributions recorded for 0.1% w/w dispersions of a series of PLMA_14_–PHEMA_*y*_ nanoparticles (*y* = 25–149) prepared by RAFT dispersion polymerization of HEMA in *n*-dodecane at 90 °C when targeting 20% w/w solids. (b) Relationship between the apparent *z*-average diameter and the PHEMA DP for the same series of nanoparticles.

**Figure 3:**
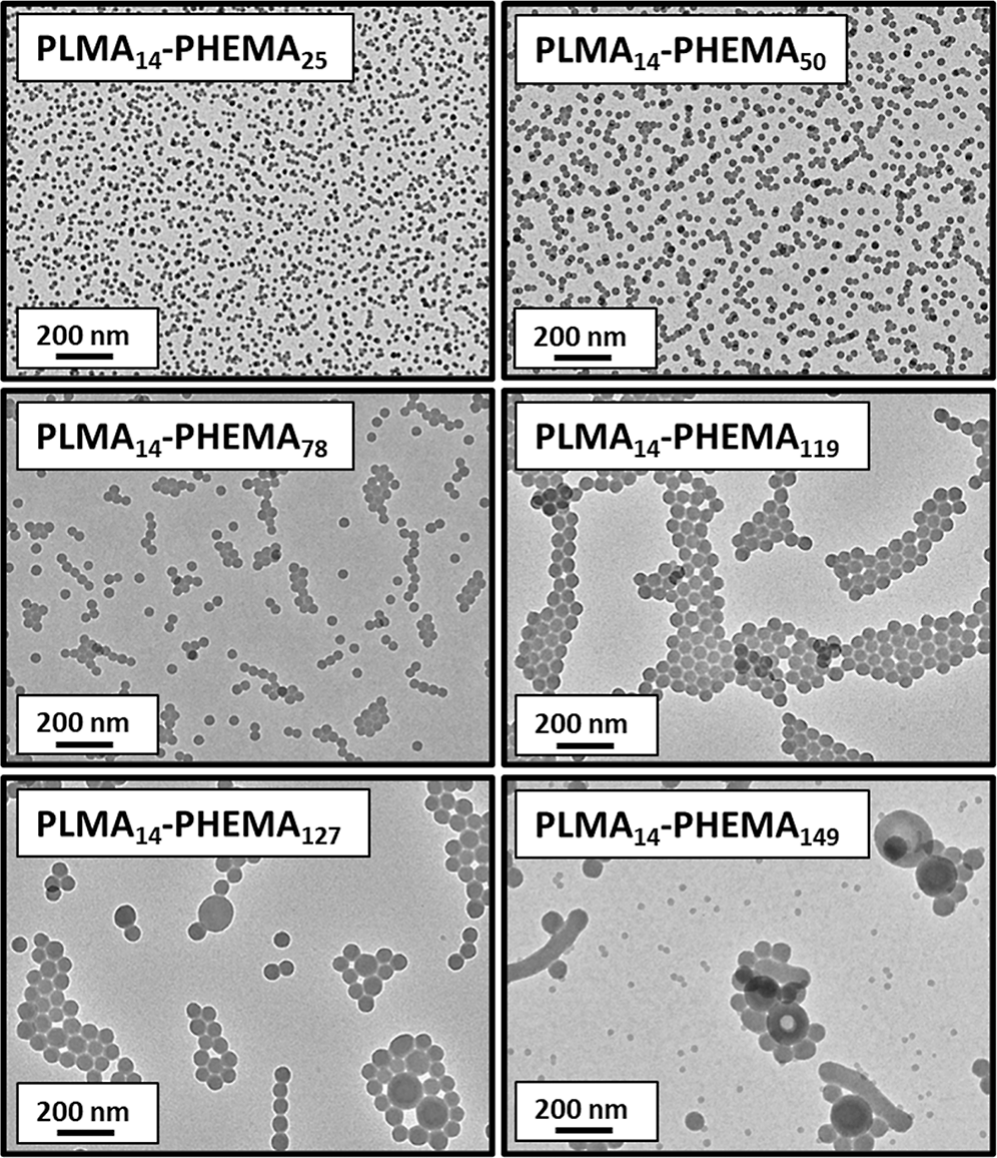
Representative TEM images recorded for selected PLMA_14_–PHEMA_*y*_ nanoparticles (where *y* = 25, 50, 78, 119, 127 and 149).

DLS analysis of the resulting diblock copolymer nanoparticles indicated a gradual increase in *z*-average particle diameter when targeting higher PHEMA DPs (see Figure 2a). A linear increase in nanoparticle diameter is observed up to a PHEMA DP of 119 (see Figure 2b). The sudden increase in apparent size observed for higher PHEMA DPs is attributed to the formation of mixed phases that contain higher order morphologies as well as spheres. This interpretation is corroborated by TEM analysis of this PLMA_14_–PHEMA_*y*_ series, which revealed that well-defined spherical nanoparticles of increasing size are obtained when targeting PHEMA DPs of 25 to 119 (see Figure 3). However, targeting a higher PHEMA DP leads to the formation of either vesicles (DP = 127) or vesicles plus short worms (DP = 149) in coexistence with a population of spheres. Perhaps surprisingly, neither a pure worm phase nor a pure vesicle phase could be obtained for such formulations, at least under the stated reaction conditions.

### SAXS Studies of Selected PLMA_14_–PHEMA_*y*_ Nanoparticles

**Figure 4:**
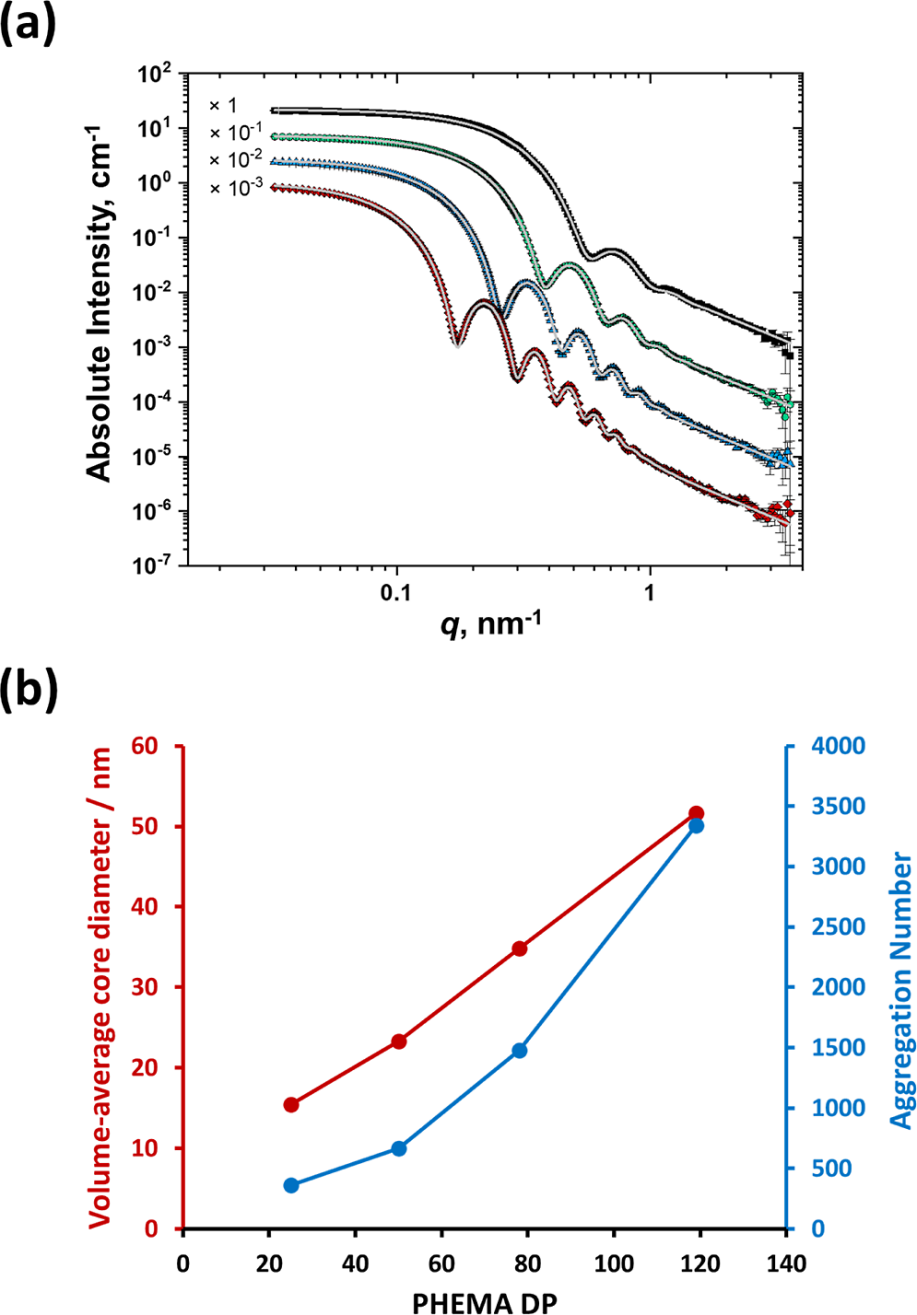
(a) SAXS patterns recorded for 1.0% w/w diblock copolymer dispersions in *n*-dodecane (PLMA_14_–PHEMA_25_, black squares; PLMA_14_–PHEMA_50_, green circles; PLMA_14_–PHEMA_78_, blue triangles; and PLMA_14_–PHEMA_119_, red diamonds) fitted using a well-known spherical micelle model (gray curves).^72^ For clarity, these SAXS patterns are offset by an arbitrary multiplication factor. (b) Variation in volume-average core diameter (red data) and mean aggregation number (blue data) with PHEMA DP for the same four examples of PLMA_14_–PHEMA_25–119_ nanoparticles.

Four examples of PLMA_14_–PHEMA_25–119_ nanoparticles were analyzed by SAXS at an international synchrotron facility. The corresponding *I*(*q*) vs *q* plots recorded for 1.0% w/w nanoparticle dispersions are shown in Figure 4a. The SAXS pattern obtained for the largest nanoparticles (PLMA_14_–PHEMA_119_) has at least six minima, which suggests the formation of near-monodisperse spheres. This is consistent with the TEM images shown in Figure 3. Applying the well-known relation *R* = 4.49/*q* (where *R* is the particle radius)^71^ to the position of the first minimum of this SAXS pattern indicates a volume-average diameter of approximately 52 nm.

For this particular diblock copolymer composition, the X-ray scattering length density contrast between the PHEMA cores and *n*-dodecane (4.36 × 10^10^ cm^–2^) is significantly greater than that between the PLMA stabilizer chains and *n*-dodecane (1.40 × 10^10^ cm^–2^). Thus, to a reasonable first approximation, the position of this first minimum corresponds to the PHEMA volume-average core diameter rather than that of the overall nanoparticles. A satisfactory fit to this SAXS pattern can be obtained using a well-known spherical micelle model,^72^ which indicates a mean aggregation number of approximately 3350 (see Supporting Information for further details). The relationship between volume-average diameter (plus mean aggregation number) and PHEMA DP for the four examples of PLMA_14_–PHEMA_25–119_ nanoparticles is shown in Figure 4b.

**Figure 5:**
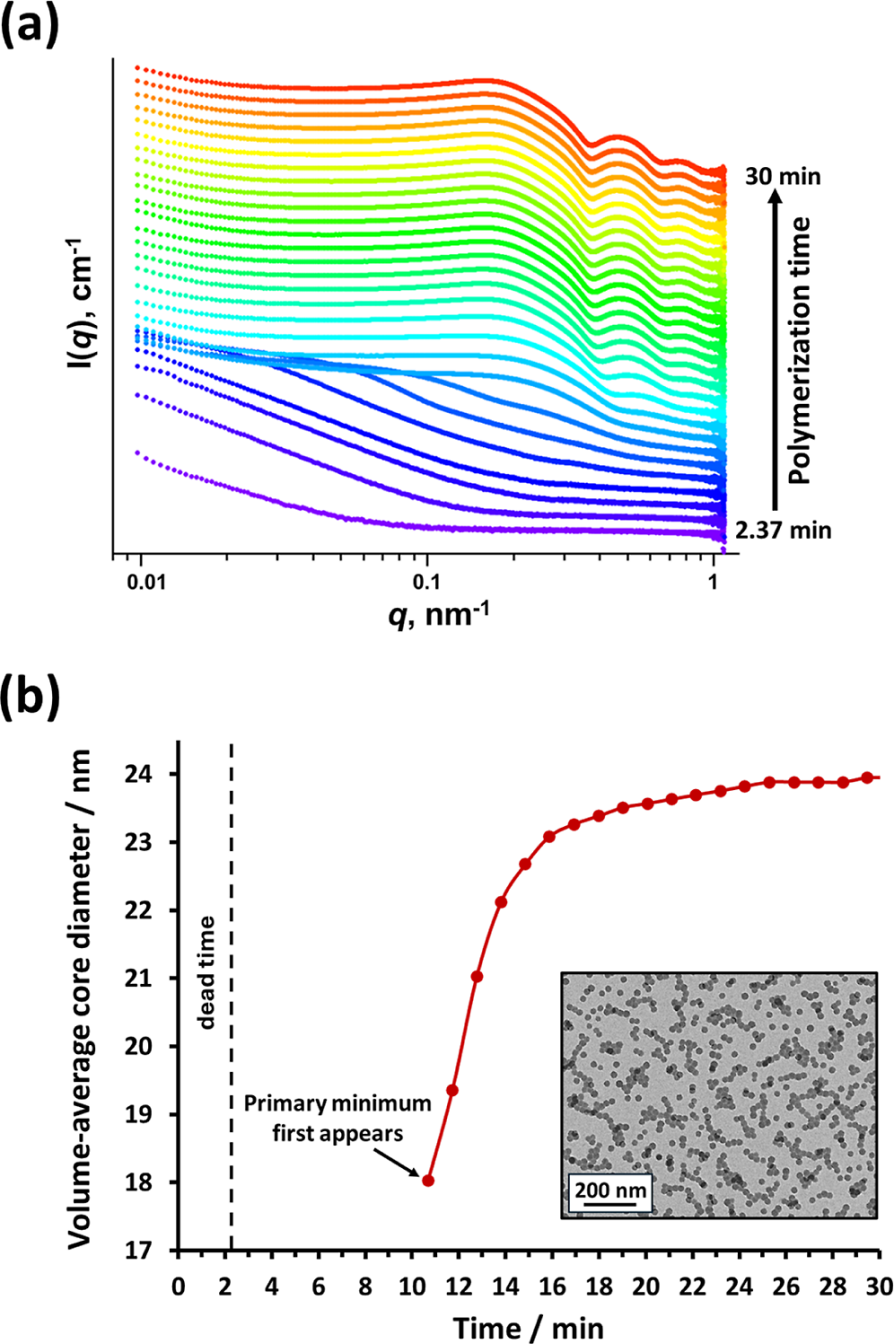
(a) Time-resolved SAXS patterns recorded during the RAFT dispersion polymerization of HEMA at 90 °C when targeting 20% w/w solids in *n*-dodecane. For clarity, each SAXS pattern has been scaled by an arbitrary factor. (b) Plot of volume-average core diameter vs reaction time for the same polymerization. This kinetic experiment had a dead time of 2.37 min (see Figure S1) and the first primary minimum was observed after 10.68 min. Inset: representative TEM image recorded for the final PLMA_14_–PHEMA_48_ nanoparticles.

One of these four nanoparticle formulations (PLMA_14_–PHEMA_50_) was selected for a time-resolved SAXS study during the RAFT dispersion polymerization of HEMA. The corresponding series of *I*(*q*) vs *q* plots is shown in Figure 5a.

This synthesis was conducted at 90 °C using a flow-through cell when targeting 20% w/w solids (see Experimental Section and Figure S1 for further details). No further change in the SAXS pattern was observed after 25 min, which indicates that the RAFT dispersion polymerization of HEMA is essentially complete within this time scale. This is consistent with observations reported for PISA syntheses conducted in non-polar media using other polar monomers.^32,63^ Again, multiple minima are observed for the final nanoparticles, indicating a relatively narrow particle size distribution. In addition, a structure factor is observed at *q* ∼ 0.15 nm^–1^ owing to interactions between neighboring nanoparticles within this relatively concentrated dispersion. Analysis of the evolution of the primary minimum in each SAXS pattern enables the increase in nanoparticle diameter to be monitored over time (see Figure 5b). The final PHEMA volume-average core diameter of around 24 nm was consistent with DLS analysis of the final nanoparticles produced in this kinetic study, which indicated a *z*-average diameter of 34 nm (DLS PDI = 0.05). TEM analysis of the final nanoparticles confirmed a well-defined spherical morphology of uniform spheres (see inset shown in Figure 5b).

### Synthesis of PLMA_196_–PHEMA_*y*_ Nanoparticles at 10% w/w Solids Using a Single Batch One-Shot Protocol

According to the PISA literature, many RAFT dispersion polymerization formulations^29,31−33,73^ yield only kinetically trapped spherical nanoparticles when using a sufficiently long precursor as the steric stabilizer block. This is undoubtedly because sphere–sphere fusion, which is the critical first step for the evolution of higher order morphologies (e.g., worms or vesicles), becomes disfavored under such conditions owing to more effective steric stabilization. Hence a longer PLMA_196_ precursor was prepared to target a series of well-defined kinetically trapped spheres and hence avoid the mixed phase problem observed when using the PLMA_14_ precursor. Accordingly, an unpurified PLMA_196_ precursor was chain-extended via RAFT dispersion polymerization of HEMA in *n*-dodecane to produce a series of PLMA_196_–PHEMA_*y*_ nanoparticles (*y* = 95–990) at 10% w/w solids. Unfortunately, the esterification protocol that enabled GPC analysis of the relatively short PLMA_14_–PHEMA_25–149_ chains did not produce molecularly dissolved copolymer chains when targeting significantly higher PHEMA DPs. Similar observations have been recently reported for an analogous aqueous PISA formulation, whereby targeting higher core-forming PHEMA DPs resulted in significantly broader molecular weight distributions when utilizing a 3:1 chloroform/methanol mixed eluent for GPC analysis.^74^ This is because the batch of triply distilled HEMA monomer that was used both in this prior study and the current manuscript has a dimethacrylate content of approximately 0.1 mol %. This inevitably leads to core cross-linking when targeting higher PHEMA DPs.^75,76^

**Figure 6:**
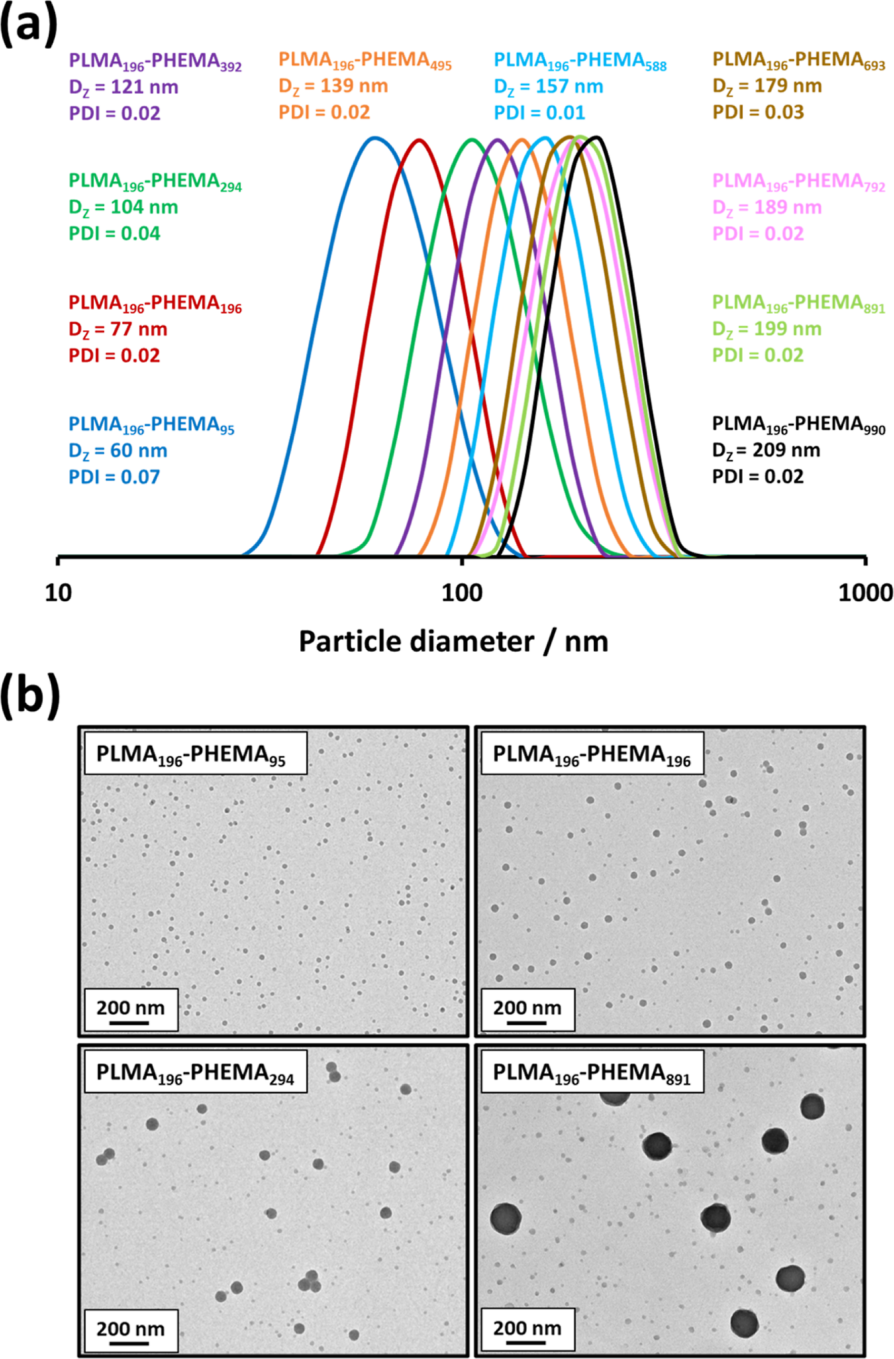
(a) DLS particle size distributions recorded for 0.1% w/w dispersions of PLMA_196_–PHEMA_*y*_ (*y* = 95–990) nanoparticles. (b) Representative TEM images recorded for selected PLMA_196_–PHEMA_*y*_ nanoparticles (*y* = 95, 196, 294 or 891).

DLS analysis of the nanoparticles indicated a gradual increase in the *z*-average particle diameter when targeting higher PHEMA DPs, as expected (see Figure 6a). However, TEM analysis revealed the unexpected presence of a bimodal size distribution above a PHEMA DP of 95, with the size difference between the two nanoparticle populations becoming progressively more pronounced (see Figure 6b). In this context, it is important to recall that DLS is strongly biased toward larger nanoparticles because they scatter light much more intensely than smaller nanoparticles. More specifically, the scattered light intensity, *I*_scat_ ∼ *R*^6^, where *R* is the mean nanoparticle radius.^77^

**Scheme 3:**
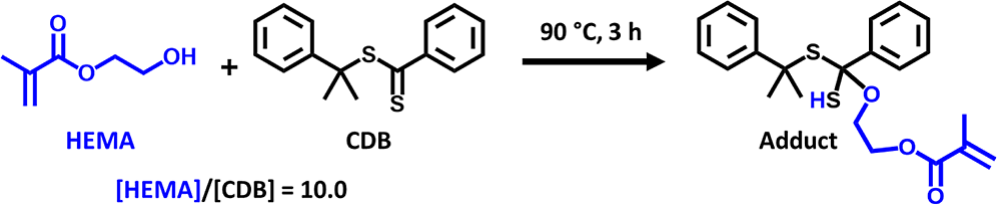
Postulated Side-Reaction between HEMA and CDB at 90 °C ^1^H NMR spectroscopy and LC–MS analysis (see Figure S4) suggest that this HEMA/CDB adduct is formed on heating a 10:1 HEMA/CDB mixture for 3 h in the absence of any initiator. A similar side-reaction is proposed to occur between HEMA and the dithiobenzoate-capped copolymer chains at 90 °C within the monomer-swollen PLMA–PHEMA nanoparticles formed during the PISA syntheses reported herein.

When we first observed bimodal particle size distributions during our TEM studies, we hypothesized that the copolymer chains within the smaller nanoparticles had prematurely lost most of their dithiobenzoate end-groups and hence had stopped growing during the HEMA polymerization.^78^ Moreover, we anticipated that UV GPC analysis should enable this tentative hypothesis to be critically examined. Accordingly, centrifugation was used to separate the large and small nanoparticles produced during the one-shot synthesis of PLMA_196_–PHEMA_1000_ nanoparticles at 10% w/w solids. Then gravimetry was used to determine the relative mass of each population. The large nanoparticles contributed 86% of the total mass, with the small nanoparticles forming the minor fraction. Centrifugal separation of these two populations enabled their subsequent esterification using excess acetyl chloride for GPC analysis. According to the data shown in Figure S3, there is a significant difference in copolymer molecular weight between the two nanoparticle populations. More specifically, the refractive index detector GPC data indicated that the larger nanoparticles comprised a higher proportion of longer copolymer chains, which is not unexpected (Figure S3a). However, UV GPC analysis indicated that there are significantly *fewer* dithiobenzoate end-groups associated with such copolymer chains compared to the shorter copolymer chains that form the relatively small nanoparticles (Figure S3b). Thus our original hypothesis was clearly incorrect. Instead, it seems that the larger nanoparticles grow at the expense of the smaller nanoparticles but also ultimately lose more of their dithiobenzoate end-groups. Given that the growing nanoparticles are swollen with HEMA, the primary alcohol group on this monomer could attack the dithiobenzoate chain-ends at 90 °C, which would lead to loss of RAFT control. To test this hypothesis, a 10:1 HEMA/CDB mixture was heated to 90 °C for 3 h in the absence of any initiator. ^1^H NMR analysis of this reaction mixture before and after heating indicated a subtle change in the aromatic signals corresponding to the CDB RAFT agent (Figure S4a). With the aid of predictive NMR software (see Figure S4b), this new signal at around 8.3 ppm was assigned to the two ortho protons on the phenyl ring for the adduct species shown in Scheme 3, which suggests that HEMA monomer does indeed react with the CDB at 90 °C. This hypothesis was corroborated by liquid chromatography–mass spectrometry (LC–MS) analysis of the HEMA/CDB reaction mixture, which provided direct evidence (see Figure S5) for the formation of this side-product. Given that the growing nanoparticles have HEMA-swollen cores, a similar side-reaction is likely to occur between HEMA and the dithiobenzoate-capped copolymer chains at 90 °C. Since this side-reaction involves the pendent primary alcohol group on this monomer, it is not surprising that no such problem is observed when conducting similar one-shot batch syntheses using alternative monomers such as benzyl methacrylate.^44^ Moreover, it should be possible to minimize this side-reaction by restricting the HEMA concentration within the growing nanoparticles at any given time (see later).

DLS analysis of the corresponding nanoparticles is also informative (see Figure S3c). The bimodal size distribution of nanoparticles with a nominal PLMA_196_–PHEMA_990_ composition has a *z*-average diameter of 209 nm and an anomalously low DLS polydispersity of 0.02. This is because the light scattering is dominated by the major population of relatively large nanoparticles, which in turn means that this technique is insensitive to the presence of the minor population of relatively small nanoparticles. Indeed, this interpretation is confirmed by DLS analysis of each of these two populations after their separation by centrifugal sedimentation. The larger nanoparticles have a *z*-average diameter of 211 nm and an associated DLS polydispersity of 0.03, which is almost identical to that obtained for the original bimodal distribution of nanoparticles. In contrast, the smaller nanoparticles have a *z*-average diameter of 64 nm and an associated DLS polydispersity of 0.09.

It is perhaps worth considering why this side-reaction has not been previously reported for other PISA formulations involving hydroxy-functional vinyl monomers and dithiobenzoate-based RAFT agents.^74,79−81^ However, it seems that in every case either the reaction temperature was significantly lower than 90 °C (e.g., 50 or 70 °C) or the monomer (e.g., HEMA or HBMA) was much less strongly partitioned within the nascent nanoparticles. One apparent exception is the RAFT dispersion polymerization of 2-hydroxypropyl methacrylate (HPMA) at 90 °C in mineral oil.^32^ However, in this case the HPMA monomer comprises two isomers, with the major isomer containing a less reactive secondary alcohol group. In the case of RAFT alcoholic dispersion polymerization,^82−85^ ethanol (or methanol) is largely excluded from the nanoparticle cores after micellar nucleation and so cannot access the dithiobenzoate end-groups. Hence this side-reaction appears to be the result of the relatively high reaction temperature and target PHEMA DP, combined with the strong partitioning of the HEMA monomer within the growing nanoparticle cores.

### Kinetic Study of the RAFT Dispersion Polymerization of HEMA at 90 °C

**Figure 7:**
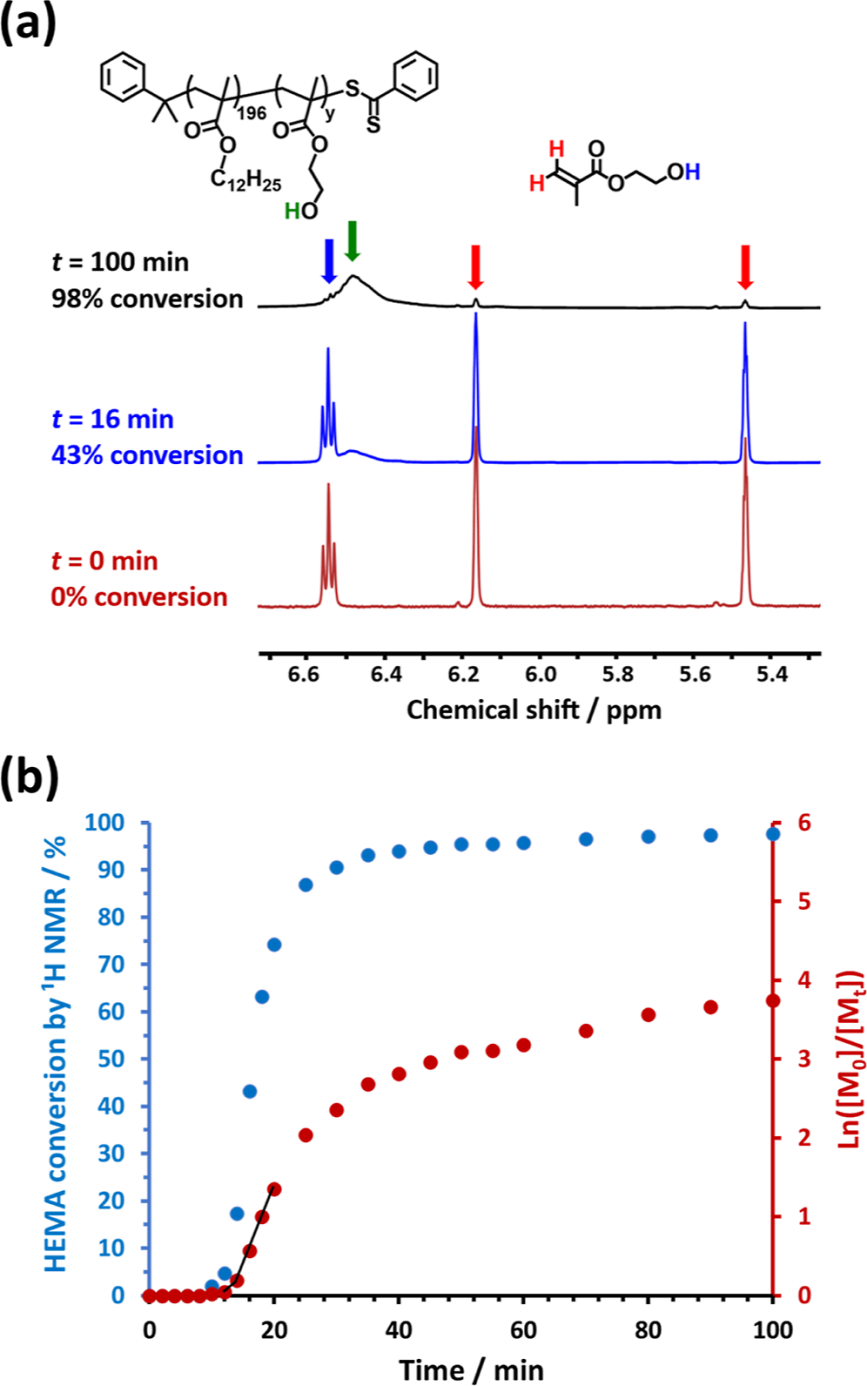
(a) Selected partial ^1^H NMR spectra recorded during the RAFT dispersion polymerization of HEMA in *n*-dodecane at 90 °C when targeting PLMA_196_–PHEMA_1000_ nanoparticles at 10% w/w solids: *t* = 0 min (red curve), *t* = 16 min (blue curve) and *t* = 100 min (black curve). (b) Conversion vs time curve (blue data) and corresponding semilogarithmic plot (red data) calculated for the same PISA formulation.

A kinetic study of the RAFT dispersion polymerization of HEMA in *n*-dodecane at 90 °C when targeting PLMA_196_–PHEMA_1000_ spherical nanoparticles at 10% w/w solids was undertaken to (i) examine the reaction time required to achieve high monomer conversion and (ii) monitor the evolution of the copolymer morphology over time. Aliquots of the reaction mixture were periodically extracted and diluted using *d*_5_-pyridine prior to analysis by ^1^H NMR spectroscopy. At each point, the fractional HEMA conversion was calculated by comparing the intensity of the HEMA monomer vinyl signals at δ 5.4 – 5.5 and δ 6.1 – 6.2 to that of the growing hydroxyl signal (HOCH_2_CH_2_−) at δ 6.5 – 6.6, as illustrated in Figure 7a. The corresponding semilogarithmic plot indicates an induction period of approximately 8 min, followed by initial slow polymerization up to 12 min (5% HEMA conversion, or a PHEMA DP of approximately 50). At this point, a 2.8-fold rate enhancement is observed, which marks the onset of micellar nucleation (see Figure 7b). The PHEMA block becomes insoluble in the reaction mixture and the resulting in situ self-assembly leads to the formation of nascent nanoparticles.^3,29,31−33,47^ A HEMA conversion of 96% is achieved within 60 min, with a relatively slow rate of polymerization being observed thereafter under monomer-starved conditions. The final monomer conversion was determined to be 98% after 100 min at 90 °C.

**Figure 8:**
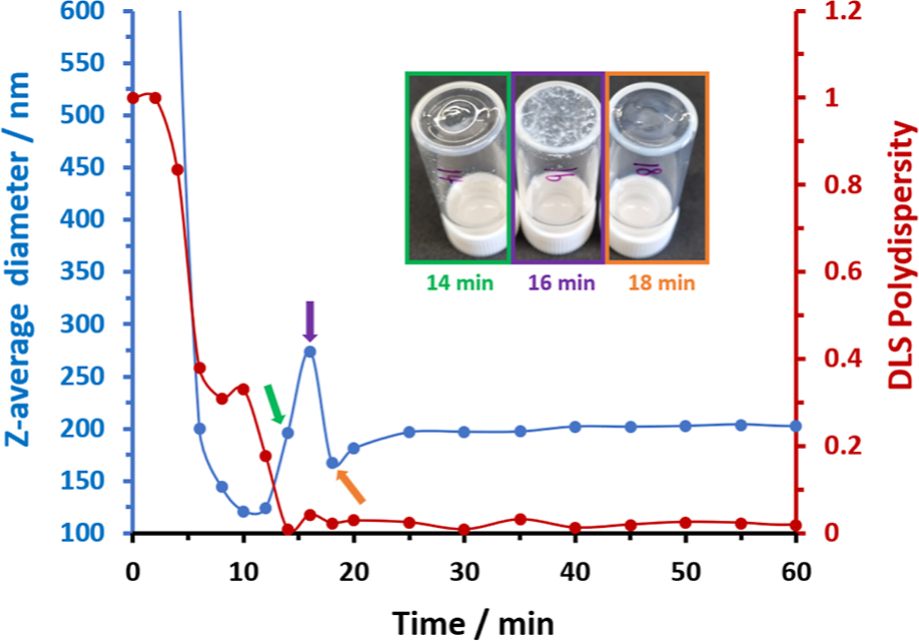
Evolution in *z*-average diameter (blue data) and DLS polydispersity (red data) over time recorded for aliquots periodically extracted from the reaction mixture when targeting PLMA_196_–PHEMA_1000_ nanoparticles using the one-shot batch protocol at 90 °C in *n*-dodecane at 10% w/w solids. Inset: digital photographs recorded to illustrate the physical appearance of aliquots extracted after 14, 16, and 18 min.

**Figure 9:**
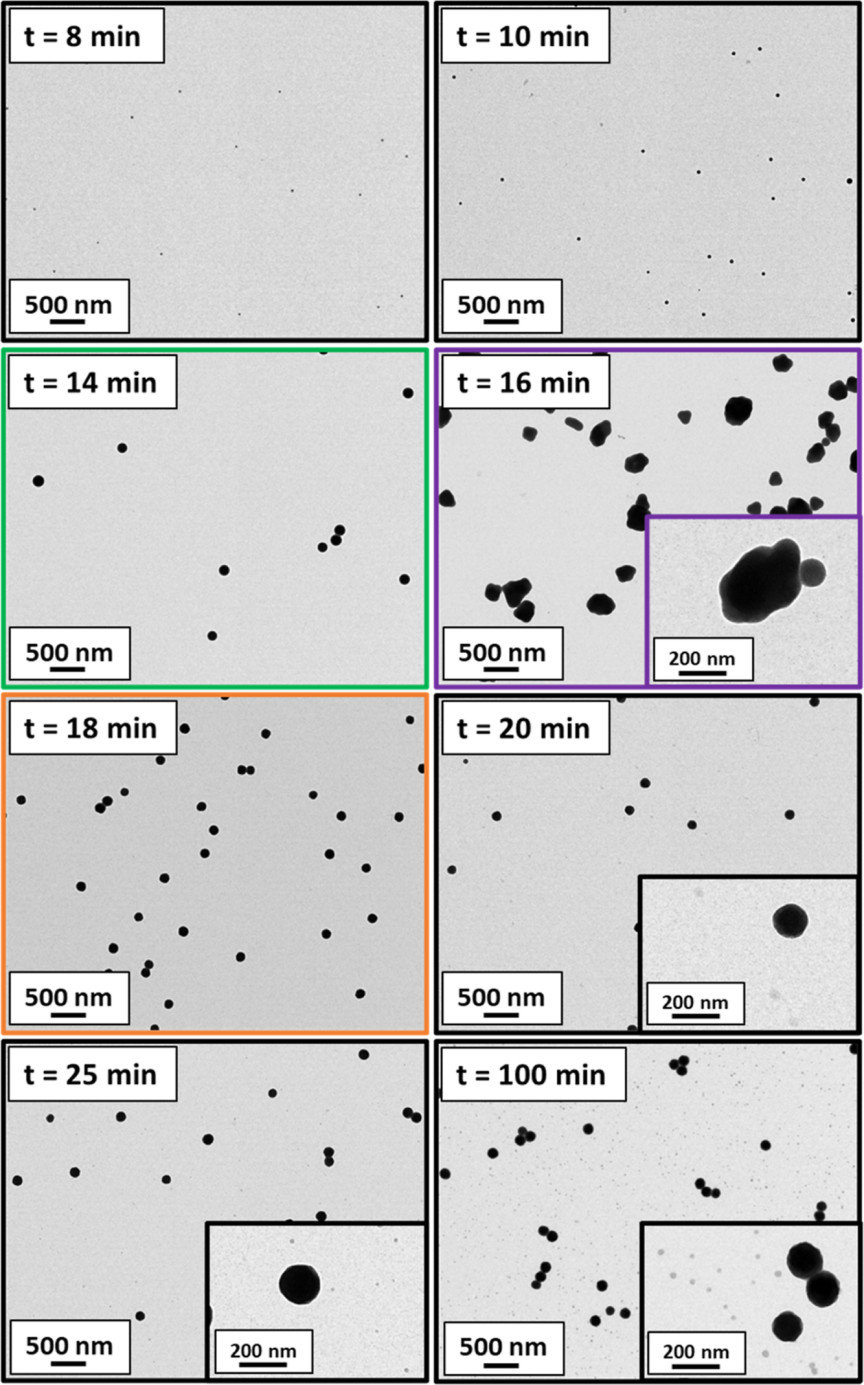
Representative TEM images recorded for aliquots extracted after 8, 10, 14, 16, 18, 20, 25, and 100 min, respectively when targeting PLMA_196_–PHEMA_1000_ nanoparticles using the one-shot batch protocol at 90 °C in *n*-dodecane at 10% w/w solids.

The aliquots extracted during this experiment were also analyzed by DLS and TEM. Each aliquot was diluted in *n*-dodecane to 0.1% w/w prior to analysis. A rapid reduction in *z*-average diameter from 0 to 10 min was observed for the prenucleation stage (see Figure 8), with the latter time point lying close to the micellar nucleation event indicated in the ^1^H NMR kinetics study of the same PISA formulation. Surprisingly, the *z*-average diameter then rapidly increases, reaching a maximum value of 274 nm after 16 min before falling to 168 nm after 18 min (see Figure 8). Subsequently, the *z*-average diameter increased gradually up to 40 min at which point, a constant value of 204 nm was observed. Selected corresponding TEM images are shown in Figure 9. The TEM image recorded after 16 min suggests the fusion of multiple spherical nanoparticles to form much larger non-spherical aggregates (see inset image). Moreover, such aggregates appear to be colloidally unstable because macroscopic precipitation is clearly visible in the inset digital photographs. Remarkably, a unimodal particle size distribution comprising relatively small nanoparticles was observed after 18 min. The TEM image recorded for the aliquot extracted after 20 min confirmed the presence of spherical nanoparticles, which grow larger as the HEMA polymerization continues. The final reaction mixture had a distinctly bimodal size distribution comprising relatively large and relatively small spherical nanoparticles. This kinetic study was repeated to assess the reproducibility of these unexpected observations: very similar DLS data and TEM images were obtained from this duplicate experiment (see Figure S6).

### Synthesis of PLMA_196_–PHEMA_1000_ Nanoparticles at 10% w/w Solids by Adding HEMA in Equal Batches

**Figure 10:**
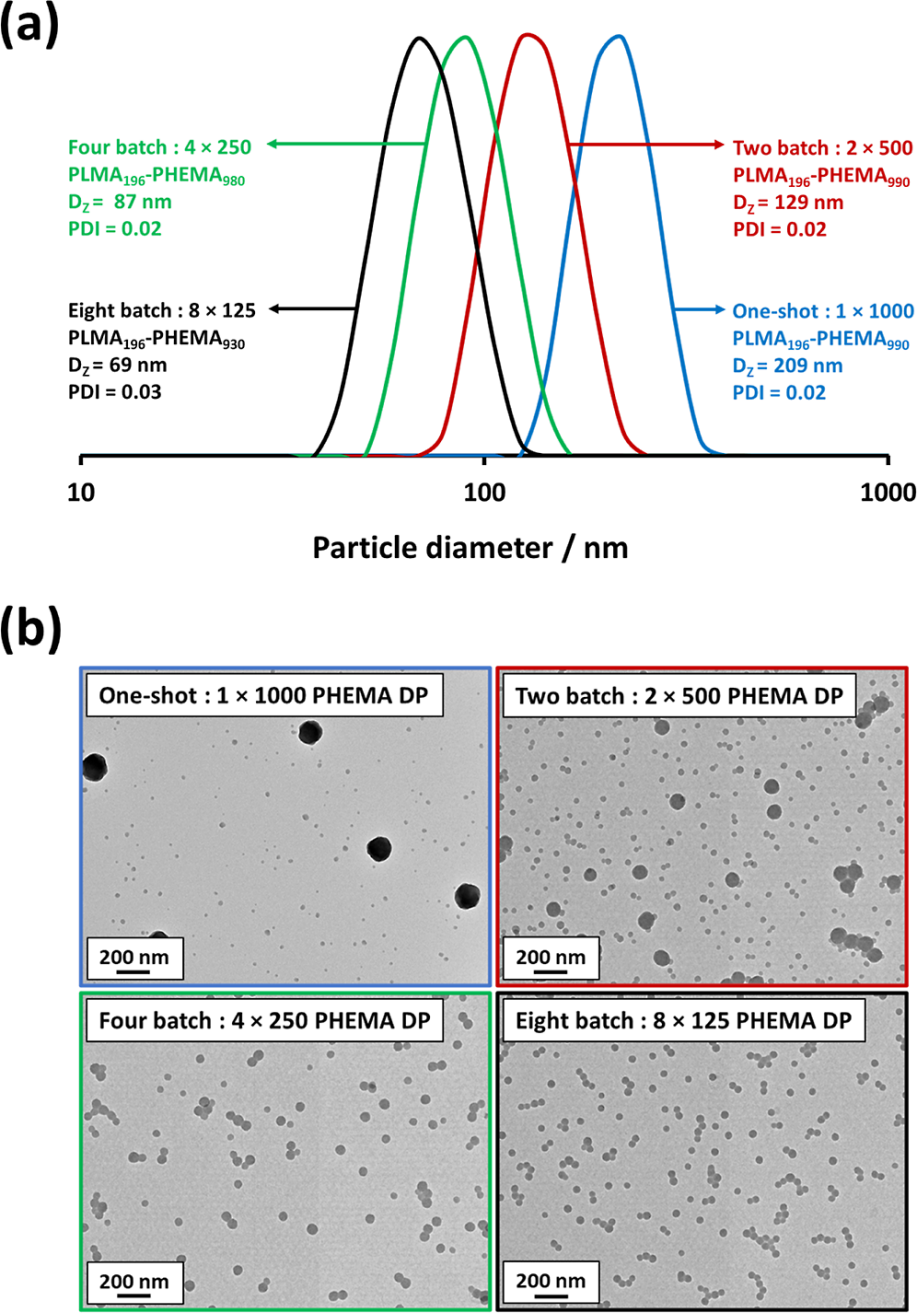
(a) DLS particle size distributions recorded for 0.1% w/w dispersions of PLMA_196_–PHEMA_1000_ nanoparticles prepared at 10% w/w solids in *n*-dodecane with HEMA monomer addition either by the one-shot batch protocol or by sequential addition of two, four or eight equal batches. (b) Representative TEM images recorded for the same nanoparticles.

To address the bimodal particle size distribution problem, HEMA monomer was added in multiple equal batches, as opposed to the with one-shot batch protocol. In principle, this should lead to a more controlled rate of polymerization and hence more uniform nanoparticle growth. For such syntheses, an unpurified PLMA_196_ precursor was chain-extended with HEMA targeting a PHEMA DP of 1000 at 10% w/w solids in *n*-dodecane by adding the HEMA in two, four or eight equal batches, with each batch being added to the reaction mixture at 1 h intervals. Based on the kinetic study (see Figure 7b), this time period was judged to be sufficient for high monomer conversion to be achieved prior to addition of the next batch. The final nanoparticle diameters were determined by DLS analysis and are shown in Figure 10a. Clearly, increasing the number of HEMA batches leads to a significant reduction in the mean particle diameter. For example, sequential addition of eight equal batches of HEMA results in an approximate three-fold reduction in nanoparticle diameter (69 nm) compared to the equivalent one-shot synthesis (209 nm). This implies that a significantly higher chain extension efficiency was achieved in the former case. This is because well-defined diblock copolymer chains self-assemble to form significantly smaller nanoparticles compared to ill-defined mixtures of polydisperse copolymer chains and homopolymer precursor chains. The corresponding TEM images recorded for such experiments are shown in Figure 10b. Again, increasing the number of HEMA batches undoubtedly leads to a significant reduction in particle size. Importantly, the eight-batch protocol produces a monomodal particle size distribution. This suggests that the bimodal particle size distribution obtained using the single batch one-shot protocol is most likely due to rapid, poorly controlled polymerization when excess HEMA monomer is present in the reaction mixture.

### Synthesis of PLMA_196_–PHEMA_*y*_ Nanoparticles under Monomer-Starved Conditions

**Figure 11:**
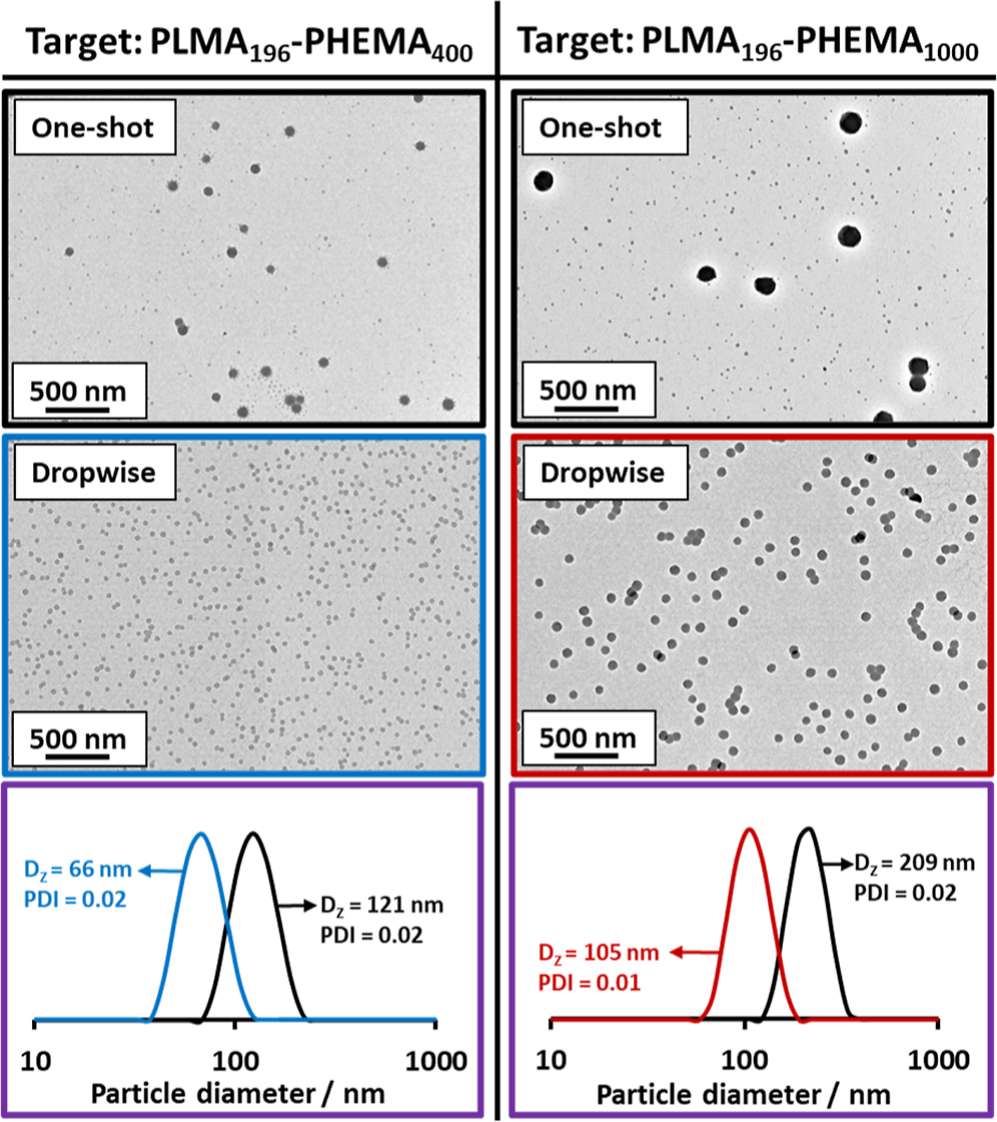
Representative TEM images recorded when targeting PLMA_196_–PHEMA_400_ and PLMA_196_–PHEMA_1000_ nanoparticles prepared by the one-shot batch protocol (black) and monomer-starved addition of HEMA monomer (blue and red). The corresponding DLS particle size distributions recorded for 0.1% w/w dispersions of PLMA_196_–PHEMA_400_ and PLMA_196_–PHEMA_1000_ nanoparticles prepared via one-shot batch addition or monomer-starved addition of HEMA are also shown (purple panels).

In view of the above observations, the PISA synthesis of PLMA_196_–PHEMA_*y*_ nanoparticles was also conducted under monomer-starved conditions, whereby HEMA monomer was continuously added to the reaction mixture over time using a motorized syringe pump. This approach is rarely utilized in the PISA literature.^86,87^ This is perhaps surprising because it is widely used for the industrial manufacture of latex particles, not least because it minimizes compositional drift when conducting statistical copolymerizations and provides good control over the reaction exotherm.^77^ In the present study, PHEMA DPs of 400 and 1000 were targeted at 10% w/w solids using the unpurified PLMA_196_ precursor. TEM images obtained for PLMA_196_–PHEMA_*y*_ nanoparticles prepared using the one-shot batch protocol are compared with those produced under monomer-starved conditions (see Figure 11). In both cases, the monomer-starved protocol produced a unimodal size distribution comprising relatively small, uniform spherical nanoparticles. In contrast, a bimodal size distribution is obtained when using the one-shot batch protocol.

Moreover, the *z*-average diameter of the small, uniform nanoparticles lies between that of the two nanoparticle populations observed in the bimodal size distribution. This suggests that the former nanoparticles are likely to comprise copolymer chains that are much closer to the target PLMA_196_–PHEMA_400_ and PLMA_196_–PHEMA_1000_ compositions. The corresponding DLS data are also shown for these four PISA syntheses. Notably, the monomer-starved protocol produced nanoparticles with a *z*-average diameter that is approximately half of that observed for the one-shot batch protocol. It is perhaps worth emphasizing here that the inherent bias of this sizing technique means that it is only sensitive to the population of larger nanoparticles when assessing bimodal size distributions.

## Conclusions

The RAFT dispersion polymerization of HEMA in *n*-dodecane using a PLMA precursor offers some interesting and unexpected challenges in terms of both synthesis and characterization. First, there are no common organic solvents that are good solvents for both PLMA and PHEMA, which complicates the GPC analysis of such diblock copolymers. Fortunately, esterification of the pendent hydroxyl groups in the PHEMA block produces derivatized copolymers that are fully soluble in chloroform. GPC analysis using this eluent indicates reasonably high blocking efficiencies for the PLMA_14_ precursor but the final copolymer molecular weight distributions are typically rather broad (*M*_w_/*M*_n_ ≥ 1.30). This is not unusual for PISA formulations in non-polar media. For this particular formulation, the known dimethacrylate impurity within HEMA monomer inevitably leads to extensive branching or cross-linking when targeting higher PHEMA DPs. Nevertheless, TEM, DLS and SAXS studies confirm that well-defined near-monodisperse spherical nanoparticles can be obtained for the PLMA_14_–PHEMA_*y*_ series. Moreover, a time-resolved SAXS experiment indicates that the RAFT dispersion polymerization of HEMA is essentially complete within 25 min at 90 °C when targeting PLMA_14_–PHEMA_50_ nanoparticles. Second, one-shot batch syntheses can lead to distinctly bimodal particle size distributions, especially when targeting higher PHEMA DPs. In our experience, such poor size control is rarely observed for PISA formulations. A control experiment suggests that HEMA most likely undergoes a hitherto unknown side-reaction with the dithiobenzoate-capped chains within the monomer-swollen nanoparticles. Fortunately, this side-reaction can be minimized either by addition of HEMA monomer in multiple batches or by its continuous addition using a syringe pump: both approaches produced well-defined spherical nanoparticles with monomodal size distributions. Third, periodic sampling of the polymerizing reaction mixture followed by DLS, TEM and NMR analysis revealed an unexpected anomaly: at around 50% HEMA conversion—which corresponds to the maximum rate of polymerization—large colloidally unstable aggregates are briefly observed that spontaneously redisperse to form well-defined colloidal nanoparticles thereafter. This remarkable behavior is unprecedented for PISA formulations and awaits a satisfactory explanation. However, our recent study^9^ suggests that this transient colloidal instability most likely coincides with the maximum local monomer concentration within the growing HEMA-swollen nanoparticles. In principle, these new spherical diblock copolymer nanoparticles offer the possibility of an interesting potential commercial application, which will be discussed in a future publication.

## Supporting Information Available

Additional tabulated GPC, DLS and TEM data obtained for various PISA formulations; schematic experimental setup used for time-resolved SAXS experiment; representative ^1^H NMR spectrum for an esterified diblock copolymer; GPC and DLS data for PLMA_196_–PHEMA_990_ nanoparticles before and after centrifugal separation of the two nanoparticle populations; ^1^H NMR spectra and LC–MS data for the HEMA/CDB control experiment; duplicate kinetic experiment for data shown in Figure 8; spherical micelle scattering model used for SAXS analysis (PDF)
