# Supplementary material for: RAFT Dispersion Polymerization of 2-Hydroxyethyl Methacrylate in Non-polar Media
Source: Macromolecules. 2024 Dec 4;57(24):11738–52. doi: 10.1021/acs.macromol.4c02016 (PMC11684172; doi:10.1021/acs.macromol.4c02016)
Supplement: Supplementary file 1 — ma4c02016_si_001.pdf [file ma4c02016_si_001.pdf]

Supporting Information for:

RAFT Dispersion Polymerization of 2-Hydroxyethyl Methacrylate in Non-Polar Media

Priyanka Chohan[1], Csilla György[1], Oleksandr O. Mykhaylyk[1], Giles M. Prentice[2], Sorin V. Filip[2], Marc J. Payne[2], Gouranga Manna[3] and Steven P. Armes[1,*]

1. Dainton Building, Department of Chemistry, University of Sheffield, Brook Hill, Sheffield, South Yorkshire, S3 7HF, UK.
2. Applied Sciences, BP Technology Centre, Whitchurch Hill, Reading, RG8 7QR, UK.
3. European Synchrotron Radiation Facility, 6 rue Jules Horowitz, 38000 Grenoble, France.

Table of Contents

Table S1. Summary of the GPC, DLS and TEM data obtained for a series of $PLMA_{14}$-$PHEMA_y$ nanoparticles prepared at 20% w/w solids in *n*-dodecane S2

Table S2. Summary of the DLS and TEM data obtained for a series of $PLMA_{196}$-$PHEMA_y$ nanoparticles prepared at 10% w/w solids in *n*-dodecane S2

Table S3. Summary of DLS and TEM data obtained for three $PLMA_{196}$-$PHEMA_{1000}$ nanoparticles prepared at 10% w/w solids in *n*-dodecane by varying the number of batches of HEMA added S3

Table S4. Summary of DLS and TEM data obtained for $PLMA_{196}$-$PHEMA_y$ nanoparticles prepared at 10% w/w solids in *n*-dodecane under monomer-starved conditions using a syringe pump S3

Figure S1. Experimental set-up used for the time-resolved SAXS study of the formation of $PLMA_{14}$-$PHEMA_{50}$ nanoparticles at 20% w/w solids in *n*-dodecane S3

Figure S2. ^1H NMR spectra recorded for a fully esterified $PLMA_{14}$-$PHEMA_{127}$ diblock copolymer and the acetyl chloride reagent S4

Figure S3. GPC curves and DLS particle size distributions for $PLMA_{196}$-$PHEMA_{990}$ nanoparticles before and after centrifugal separation of the two nanoparticle populations. S5

Figure S4. ^1H NMR spectra recorded before and after heating cumyl dithiobenzoate (CDB) with HEMA monomer at 90°C for 3 h S6

Figure S5. LC-MS data recorded after heating cumyl dithiobenzoate (CDB) with HEMA monomer at 90 °C for 3 h S7

Figure S6. Duplicate kinetic data for the synthesis of of $PLMA_{196}$-$PHEMA_{1000}$ nanoparticles at 10% w/w solids in *n*-dodecane S8

Spherical micelle scattering model used for SAXS analysis S9

Table S1. Summary of the GPC, DLS and TEM data obtained for a series of $PLMA_{14}$-$PHEMA_y$ nanoparticles prepared at 20% w/w solids in *n*-dodecane.

Target Composition	Chloroform GPC		DLS		TEM Morphology
	M_n / g mol^{-1}	M_w/M_n	D_z / nm	PDI	
$PLMA_{14}$-$PHEMA_{25}$	10,200	1.31	25	0.07	Spheres
$PLMA_{14}$-$PHEMA_{30}$	11,400	1.33	26	0.03	Spheres
$PLMA_{14}$-$PHEMA_{50}$	14,900	1.42	32	0.03	Spheres
$PLMA_{14}$-$PHEMA_{70}$	19,300	1.49	40	0.04	Spheres
$PLMA_{14}$-$PHEMA_{80}$	21,100	1.58	45	0.05	Spheres
$PLMA_{14}$-$PHEMA_{90}$	22,700	1.61	48	0.03	Spheres
$PLMA_{14}$-$PHEMA_{110}$	26,000	1.70	59	0.02	Spheres
$PLMA_{14}$-$PHEMA_{120}$	27,700	1.80	63	0.01	Spheres
$PLMA_{14}$-$PHEMA_{130}$	29,400	1.79	93	0.09	Mixed
$PLMA_{14}$-$PHEMA_{150}$	34,000	1.97	131	0.07	Mixed

Table S2. Summary of the DLS and TEM data obtained for a series of $PLMA_{196}$-$PHEMA_y$ nanoparticles prepared at 10% w/w solids in *n*-dodecane.

Target Composition	DLS		TEM Morphology
	D_z / nm	PDI	
$PLMA_{196}$-$PHEMA_{100}$	60	0.07	Spheres
$PLMA_{196}$-$PHEMA_{200}$	77	0.02	Spheres
$PLMA_{196}$-$PHEMA_{300}$	104	0.04	Spheres
$PLMA_{196}$-$PHEMA_{400}$	121	0.02	Spheres
$PLMA_{196}$-$PHEMA_{500}$	139	0.02	Spheres
$PLMA_{196}$-$PHEMA_{600}$	157	0.01	Spheres
$PLMA_{196}$-$PHEMA_{700}$	179	0.03	Spheres
$PLMA_{196}$-$PHEMA_{800}$	189	0.02	Spheres
$PLMA_{196}$-$PHEMA_{900}$	199	0.02	Spheres
$PLMA_{196}$-$PHEMA_{1000}$	209	0.02	Spheres

Table S3. Summary of DLS and TEM data obtained for three $PLMA_{196}$-$PHEMA_{1000}$ nanoparticles prepared at 10% w/w solids in *n*-dodecane by varying the number of batches of HEMA monomer.

Target Composition	Number of batches of HEMA monomer added	DLS		TEM Morphology
		D_z / nm	PDI	
$PLMA_{196}$-$PHEMA_{1000}$	2	129	0.02	Spheres
$PLMA_{196}$-$PHEMA_{1000}$	4	87	0.02	Spheres
$PLMA_{196}$-$PHEMA_{1000}$	8	69	0.03	Spheres

Table S4. Summary of DLS and TEM data obtained for $PLMA_{196}$-$PHEMA_y$ nanoparticles prepared at 10% w/w solids in *n*-dodecane under monomer-starved conditions using a syringe pump.

Target Composition	DLS		TEM Morphology
	D_z / nm	PDI	
$PLMA_{196}$-$PHEMA_{400}$	66	0.02	Spheres
$PLMA_{196}$-$PHEMA_{1000}$	105	0.01	Spheres

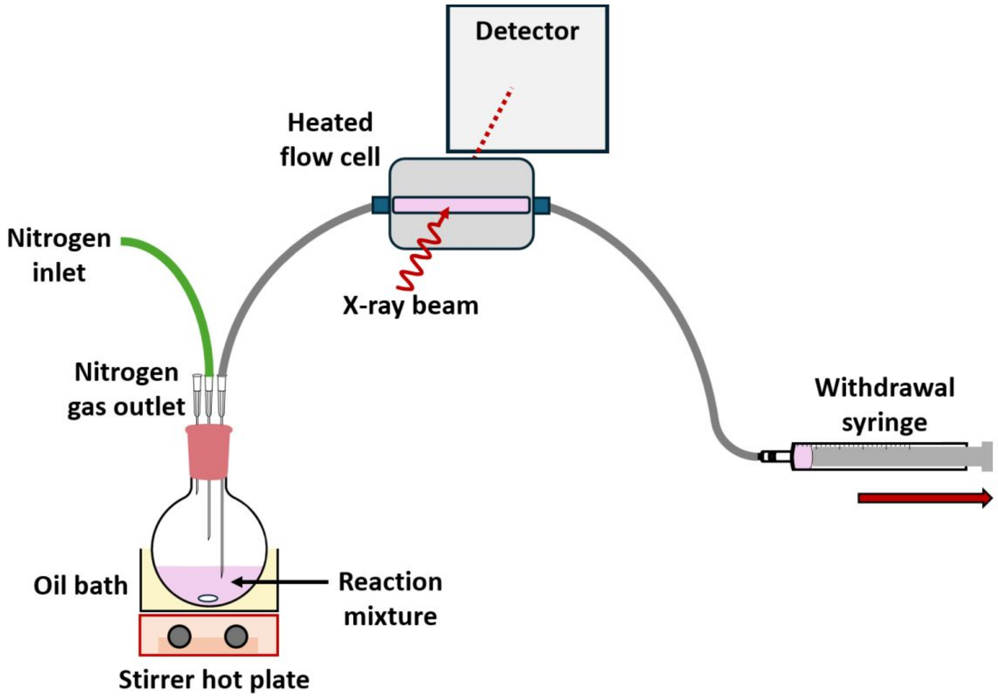

Figure S1. Schematic showing the experimental set-up for the collection of time-resolved SAXS patterns during the RAFT dispersion polymerization of HEMA targeting $PLMA_{14}$-$PHEMA_{50}$ nanoparticles at 20% w/w solids in *n*-dodecane at 90 °C.

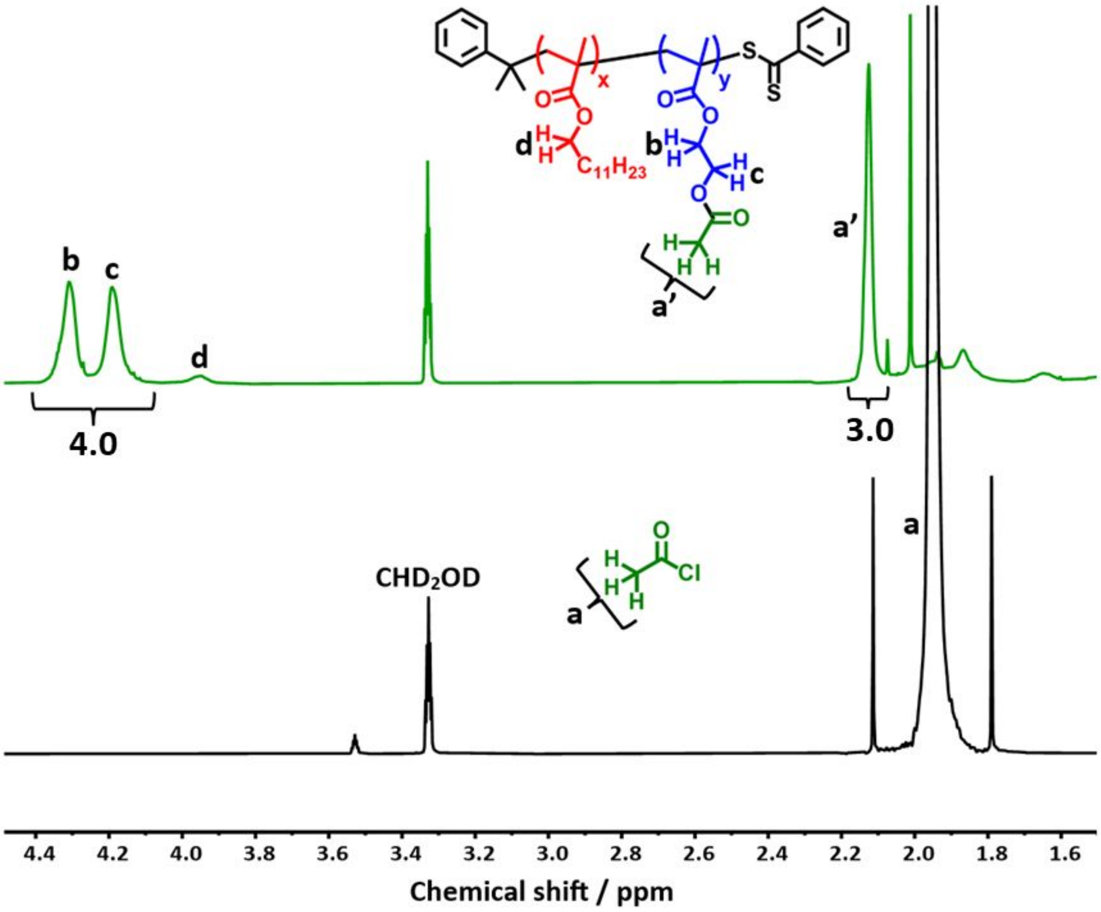

Figure S2. ^{1}H NMR spectra recorded using a 50:50 CD_3OD/CD_2Cl_2 solvent mixture for a fully esterified $PLMA_{14}$-$PHEMA_{127}$ diblock copolymer (upper green spectrum) and acetyl chloride alone (lower black spectrum). After copolymer derivatization using a $[CH_3COCl]/[HEMA]$ molar ratio of 1.5 at 25 °C in *n*-dodecane, a new signal *a'* appears at 2.1 ppm that is assigned to the acetylated HEMA repeat units. Comparing the intensity of this signal to that of the four oxymethylene protons (*b*, *c*) at 4.0 – 4.4 ppm indicates a mean degree of esterification of approximately 100%.

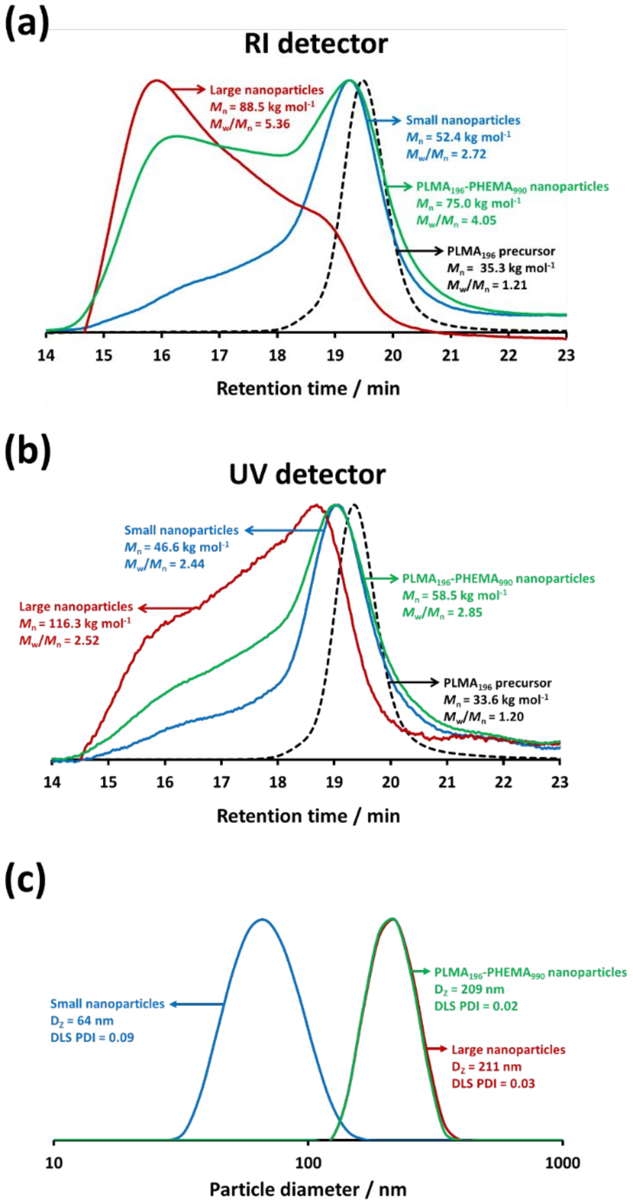

Figure S3. (a) Chloroform GPC curves (*vs*. a series of near-monodisperse poly(methyl methacrylate) calibration standards) recorded using a refractive index detector for the $PLMA_{196}$ precursor (black dashed curve) prepared by RAFT solution polymerization of LMA in *n*-dodecane at 80% w/w solids, the $PLMA_{196}$-$PHEMA_{990}$ diblock copolymer prepared by RAFT dispersion polymerization of HEMA in *n*-dodecane at 90 °C targeting 10% w/w solids (this PISA formulation afforded a distinctly bimodal particle size distribution; green curve) and the copolymer chains associated with the small (blue curve) and large (red curve) nanoparticles within the same formulation. **(b)** Chloroform GPC curves (*vs*. a series of near-monodisperse polystyrene calibration standards with M_p ranging from 2,590 to 371,100 g mol^{-1}) recorded using a UV detector set at 298 nm for the same copolymers. In all three cases, these copolymers were fully acetylated using a [CH_3COCl]/[HEMA] molar ratio of 3.0. **(c)** DLS particle size distributions recorded for the as-synthesized $PLMA_{196}$-$PHEMA_{990}$ nanoparticles (green curve) and the relatively small (blue curve) and relatively large (red curve) nanoparticles isolated from the same formulation after centrifugal sedimentation of the latter population.

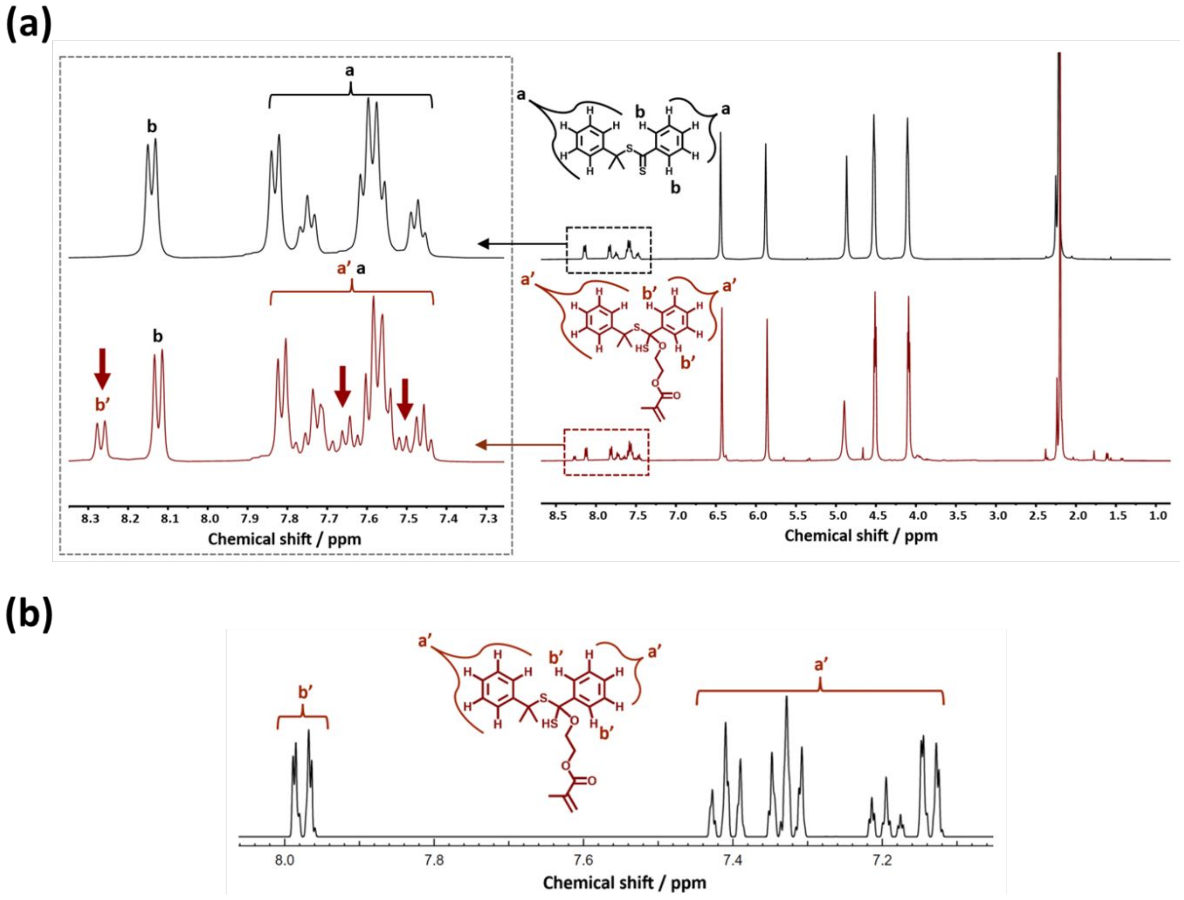

Figure S4. (a) ^{1}H NMR spectra recorded before (black spectrum) and after (red spectrum) the reaction of CDB RAFT agent with HEMA monomer at 90 °C for 3 h at a [HEMA]/[CDB] molar ratio of 10. After heating, a subtle change in the CDB signals is observed between 7.4 – 8.3 ppm. This is attributed to a side-reaction between CDB and HEMA (see labeled red chemical structure). **(b)** Partial ^{1}H NMR spectrum (aromatic region only) predicted for this adduct using open source NMR software (see https://www.nmrdb.org/about/).

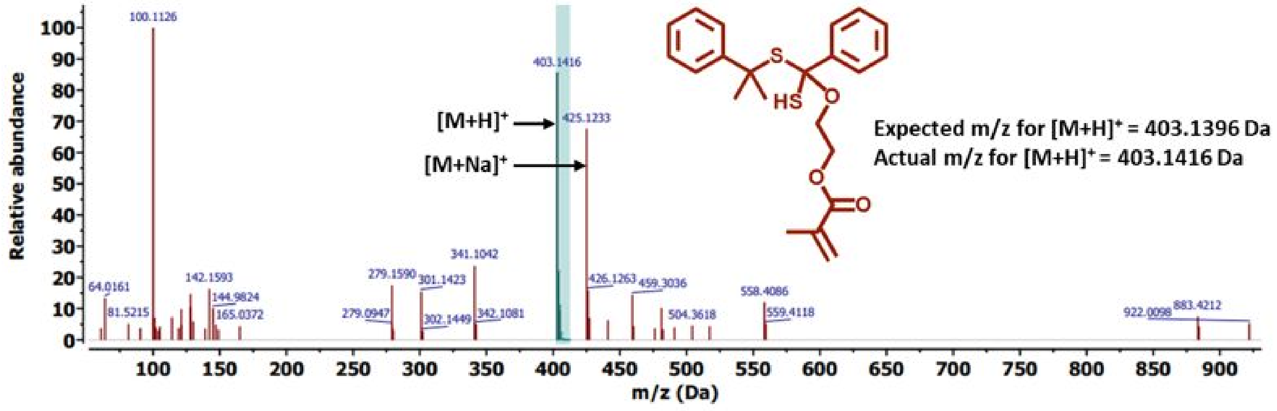

Figure S5. Liquid chromatography-mass spectrum obtained for the minor species observed at 11.67 min after heating HEMA and CDB (HEMA/CDB molar ratio = 10) at 90 °C for 3 h. This compound has an $[M+H]^+$ m/z signal at 403.1416 Da (see blue shaded area) and hence corresponds to the HEMA-CDB adduct (expected $[M+H]^+$ m/z signal = 403.1396 Da) formed by the side-reaction shown in **Scheme 3** (see main manuscript). The corresponding $[M+Na]^+$ signal is also observed at m/z = 425.1233 Da.

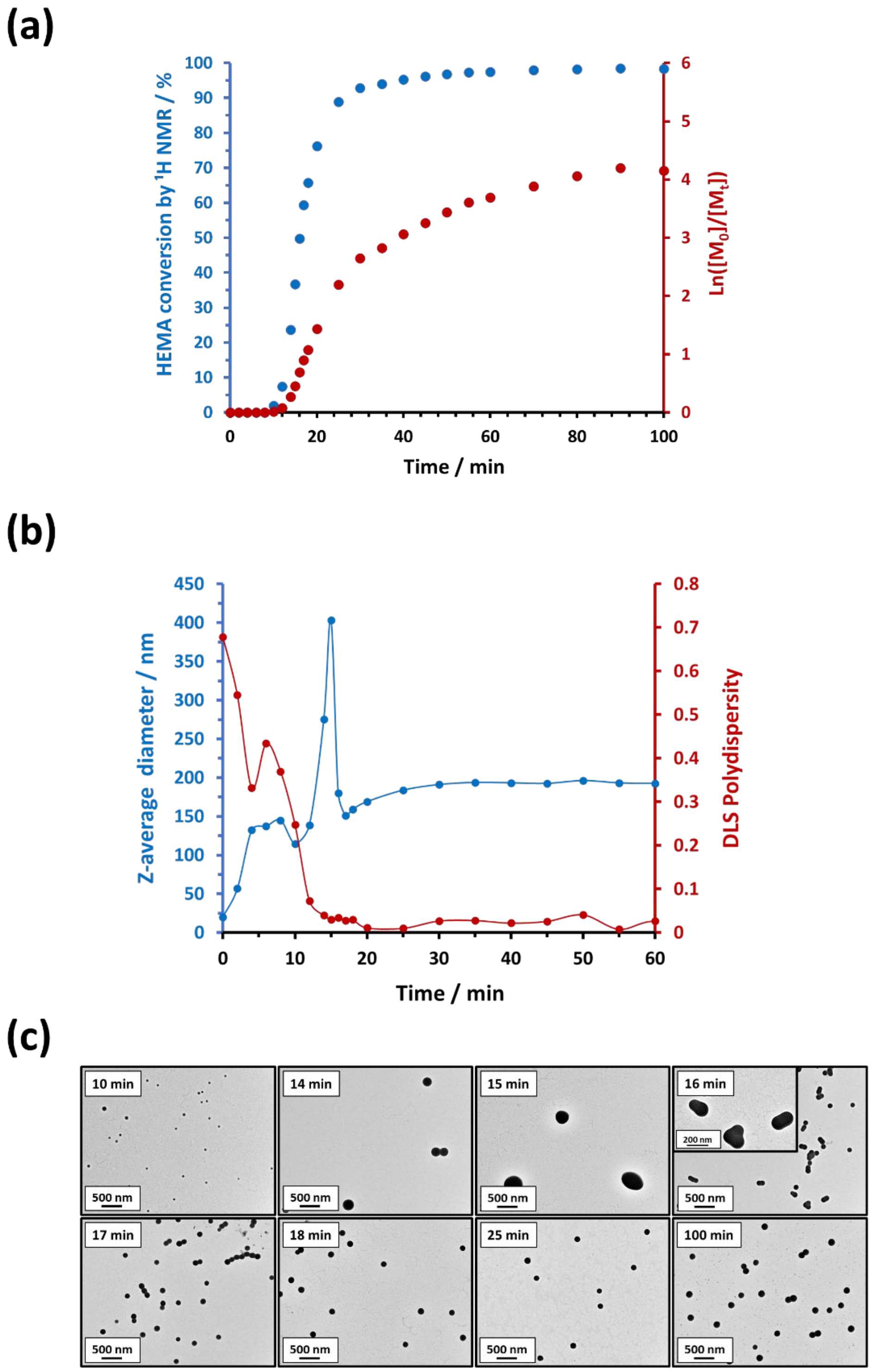

Figure S6. Duplicate kinetic study to demonstrate reproducibility for **(a)** the conversion *vs.* time curve (blue data) and corresponding semi-logarithmic plot (red data) for the RAFT dispersion polymerization of HEMA targeting $PLMA_{196}$-$PHEMA_{1000}$ nanoparticles at 10% w/w solids in *n*-dodecane at 90 °C. **(b)** Evolution in z-average diameter vs. time (blue data) and DLS polydispersity *vs.* time (red data) obtained for the periodically sampled reaction mixture. **(c)** Representative TEM images obtained for aliquots extracted from the reaction mixture after 10, 14, 15, 16, 17, 18, 25 and 100 min.

Spherical micelle scattering model used for SAXS analysis

In general, the scattering intensity of a single population of non-interacting particles can be expressed as:

$$I(q) = N\int_0^\infty ...\int_0^\infty F(q,r_1,...,r_k)^2 \Psi(r_1,...,r_k) dr_1...dr_k \quad \text{(S1)}$$

where $F(q,r_1,...,r_k)$ is the particle form factor expressed as a function of $r_1,...,r_k$ parameters, $\Psi(r_1,...,r_k)$ is the distribution function of the form factor parameters and N is the particle number density per unit volume. For this spherical micelle scattering model, only one structural parameter, the micellar core radius (r_1), is assumed to have a polydispersity, which is described by the following Gaussian distribution:

$$\Psi(r_1) = \frac{1}{\sqrt{2\pi\sigma_{Rs}^2}} e^{-\frac{(r_1 - R_s)^2}{2\sigma_{Rs}^2}} \quad \text{(S2)}$$

where R_s is the mean radius of the spherical micelle core and σ_{Rs} is its standard deviation. For a population of spherical diblock copolymer micelles, the form factor in eq. S1 can be expressed as:[1,2]

$$\begin{aligned} F_{mic}(q,r_1) &= N_{agg}^2(r_1)\beta_s^2 A_s^2(q,r_1) + N_{agg}(r_1)\beta_c^2 F_c(q,R_g) + \\ &+ N_{agg}(r_1)[N_{agg}(r_1)-1]\beta_c^2 A_c^2(q,r_1) + 2N_{agg}^2(r_1)\beta_s\beta_c A_s(q,r_1)A_c(q,r_1) \end{aligned} \quad \text{(S3)}$$

where r_1 is the micelle core radius, R_g is the radius of gyration of the corona block (PLMA), and $\beta_s = V_s(\xi_s - \xi_{sol})$ and $\beta_c = V_c(\xi_c - \xi_{sol})$ are the X-ray scattering length density contrasts for the PHEMA core block and the PLMA corona block, respectively. ξ_s, ξ_c, and ξ_{sol} are X-ray scattering length densities for the core block ($\xi_{PHEMA} = 11.68 \times 10^{10}$ cm^{-2}), the corona block ($\xi_{PLMA} = 8.72 \times 10^{10}$ cm^{-2}) and the solvent ($\xi_{n\text{-dodecane}} = 7.32 \times 10^{10}$ cm^{-2}), respectively. Finally, V_s and V_c are the volumes of the core block and the corona block, respectively. Given the mean number of repeat units in the core block and the corona block, the repeat unit molecular weight of each block (254.3 kg mol^{-1} for PLMA and 130.1 kg mol^{-1} for PHEMA), and the mass density of each block ($\rho_{PHEMA} = 1.28$ g cm^{-3}, as measured by helium pycnometer for PHEMA homopolymer, and $\rho_{PLMA} = 0.921$ g cm^{-3}, as measured by liquid densitometer measurements of a range of PLMA concentrations dissolved in *n*-dodecane), the core block and the corona block volumes can be calculated using the equation $V = \frac{M_w}{N_A \rho}$.

The mean aggregation number of the micelles is given by $N_{agg}(r_1) = (1 - x_{sol})\frac{4\pi r_1^3}{3V_s}$, where x_{sol} is the solvent concentration within the PHEMA cores. Given that HEMA monomer is not miscible with *n*-dodecane at 20°C, this solvent must be a bad solvent for the PHEMA chains. x_{sol} was set to zero for SAXS analysis. The amplitude of the core self-term is expressed as:

$$A_s(q, r_1) = \Phi(qr_1)\exp\left(-\frac{q^2\sigma^2}{2}\right) \tag{S4}$$

where $\Phi(qr_1) = \frac{3[\sin(qr_1) - qr_1\cos(qr_1)]}{(qr_1)^3}$ is the form factor amplitude of a sphere.

The exponent term in eq. S4 represents a sigmoidal interface between the blocks with a width σ with a decaying scattering length density at the core surface. This σ value was fixed at 0.22 nm during SAXS data fitting. The self-correlation term for the corona block is given by the Debye function:

$$F_c(qR_g) = \frac{2[\exp(-q^2R_g^2) - 1 + q^2R_g^2]}{q^4R_g^4} \tag{S5}$$

For certain diblock copolymer compositions (e.g. $PLMA_{14}$-$PHEMA_{25}$), the X-ray scattering contribution arising from the spherical micelle corona is comparable to that from the micelle cores [e.g. $(\beta_c / \beta_s)^2 \approx 0.2$]. In such cases, the amplitude of the corona chain form factor is obtained from a normalized Fourier transform of the radial density distribution function of the corona chains:

$$A_c(q, r_1) = \frac{\int_{r_1}^{r_1+2s} \mu_c(r)\frac{\sin(qr)}{qr}r^2dr}{\int_{r_1}^{r_1+2s} \mu_c(r)r^2dr}\exp\left(-\frac{q^2\sigma^2}{2}\right) \tag{S6}$$

The radial profile, $\mu_c(r)$, can be expressed by a linear combination of two cubic b splines, with two fitting parameters *s* and *a* corresponding to the width of the profile and the function weight coefficient, respectively. This information can be found elsewhere,[1,2] along with the approximate integrated form of eq. S6. Finally, *N* in eq. S1 is expressed as:

$$N = \frac{\phi}{\int_0^\infty V(r_1)\Psi(r_1)dr_1} \tag{S7}$$

where ϕ is the total volume fraction of diblock copolymer and $V(r_1)$ is the total volume occupied by each block within a spherical micelle, $V(r_1) = (V_s + V_c)N_{agg}(r_1)$.

[N.B. The experimental radius of gyration, R_g, determined for the PLMA stabilizer chains block using the above spherical micelle model is approximately 1.2 nm. The length of a single polymerized LMA repeat unit is 0.255 nm (i.e. two C-C bonds in an *all-trans* conformation), so the overall contour length of a $PLMA_{14}$ block, L_{PLMA} = 14 × 0.255 nm = 3.57 nm. Assuming a mean Kuhn length of 1.53 nm (based on the known literature value for PMMA), the unperturbed radius of gyration, R_g = (3.57 nm × 1.53 nm / 6)$^{0.5}$, or 0.95 nm. This theoretical R_g value does not account for the relatively bulky lauryl side-chains yet lies close to the experimental value of 1.2 nm].

References

1. J. S. Pedersen and M. C. Gerstenberg, *Colloids and Surfaces A -Physicochemical and Engineering Aspects*, 2003, **213**, 175-187.
2. J. S. Pedersen, C. Svaneborg, K. Almdal, I. W. Hamley and R. N. Young, *Macromolecules*, 2003, **36**, 416-433.
